# A Network-Medicine Framework for Intra-Oral Comorbidity: Age-Stratified Clustering and Quasi-Causal Progression Modeling from Outpatient Electronic Health Records

**DOI:** 10.3390/bioengineering13070761

**Published:** 2026-06-29

**Authors:** Wei Chen, Peng Huang, Zijian Cheng, Yaowu Chen, Xiang Tian, Yumeng Song, Xiaoyan Chen, Qianming Chen, Rui Zhang

**Affiliations:** 1 Stomatology Hospital, School of Stomatology, Zhejiang University School of Medicine, Zhejiang Provincial Clinical Research Center for Oral Diseases, Zhejiang Key Laboratory of Oral Biomedical, Hangzhou 310000, China; chenwei@zjkq.com.cn (W.C.); hp@zjkq.com.cn (P.H.);; 2 Zhejiang Provincial Key Laboratory of Internet Multimedia Technology, College of Biomedical Engineering & Instrument Science, Zhejiang University, Hangzhou 310027, China

**Keywords:** comorbidity network, oral diseases, network medicine, ICD-10, age stratification, disease progression, mediation analysis, electronic health records, real-world data, biomedical informatics

## Abstract

Background: Network medicine has reshaped how systemic comorbidities are quantified, but the internal comorbidity structure of oral diseases remains undescribed at four-character ICD-10 granularity. Methods: A total of 2,863,671 outpatient visit records from 583,614 patients (2011–2025) were analyzed. Using ICD-10 four-character codes (75 disease nodes), comorbidity networks were constructed for five age strata, with edges selected by relative risk (RR) > 1.5 and Bonferroni-corrected Fisher’s exact tests. Patient-level longitudinal sequences were mined for progression trajectories, and quasi-causal analyses—Cox regression, negative outcome controls, and Baron–Kenny mediation—were used to evaluate pathway directionality and specificity. Results: The all-age network contained 75 nodes and 167 edges (modularity = 0.53), forming eight communities. Network complexity peaked at 18–29 years and declined with age. Dental caries emerged as the strongest hub in the 60+ stratum (degree = 9). Cox regression adjusted for age, sex, and healthcare utilization confirmed pathway directionality (pulpitis → tooth defect: hazard ratio (HR) = 2.65; caries → pulpitis: HR = 2.25), and negative outcome controls confirmed biological specificity. Mediation analysis showed that pulpitis completely mediated the caries → tooth defect association (proportion mediated ≈ 100%; 95% confidence interval (CI), 90–128%). An oral mucosal immune cluster (burning mouth syndrome, lichen planus, candidiasis, and xerostomia) emerged as a clinically actionable community. Conclusions: Oral diseases form biologically coherent, age-evolving comorbidity communities, and pulpitis is the critical mediating intervention point in the caries-to-tooth-defect cascade. The framework provides a reusable network-medicine substrate for age- and sex-specific risk-stratified oral disease management.

## 1. Introduction

Comorbidity refers to the co-occurrence of two or more diseases in the same patient, either simultaneously or sequentially. Traditional comorbidity research evaluates associations between diseases pairwise, an approach that fails to capture systemic interactions among multiple diseases. Network science has introduced a novel perspective: a large-scale human disease comorbidity network was first constructed using the U.S. Medicare database, revealing systematic co-occurrence patterns that exceed random expectations [1]. Subsequently, network medicine has emerged as a key paradigm for studying complex inter-disease relationships [2,3], with multilayer approaches tracking disease evolution across the lifespan [4,5] and electronic health record (EHR) mining serving as a primary route for data-driven discovery [6]. Disease-associated proteins have been shown to cluster in localized neighborhoods within the interactome, with overlapping network modules predicting disease co-occurrence [3]; a human symptoms–disease network has been constructed, demonstrating that phenotypic similarity can complement molecular evidence [7]. A large-scale comorbidity network dataset covering 8.9 million inpatients has also recently been released [8]. Collectively, these studies demonstrate that network methods can reveal systematic inter-disease associations—disease clustering, hub identification, and progression-pathway prediction—that traditional pairwise epidemiology cannot uncover.

Despite these advances in systemic network medicine, investigation into oral diseases has remained largely limited to their associations with systemic conditions. A comorbidity network between periodontal disease and systemic diseases has been constructed using UK Biobank data [9]; dental caries has been identified as a hub node in the chronic disease network [10]; the evidence linking oral health and systemic non-communicable diseases has been systematically reviewed [11]; and the multimorbidity of periodontal disease with systemic conditions has been confirmed from both clinical and community perspectives [12,13]. A common feature of these studies is that the oral disease coding is very coarse, typically using only K02 (dental caries) and K05 (periodontal disease) as two three-character nodes. As a result, the internal comorbidity structure of oral diseases—for example, the complex associations among dental caries and pulp diseases, periodontal disease and tooth loss, and oral mucosal diseases—has never been systematically described. Moreover, computational approaches to oral disease in the bioengineering literature have so far operated predominantly at the image level—for example, deep-learning recognition of periodontitis, dental caries, and periapical lesions on dental radiographs [14,15]—rather than at the population level, leaving the intra-oral comorbidity structure itself uncharacterized.

Characterizing this internal structure carries substantial bioengineering and clinical value: oral diseases affected approximately 3.69 billion people worldwide in 2021 [16], with global costs exceeding US$ 540 billion [17]. Oral diseases have well-known clinical progression relationships—caries can progress to pulpitis, periapical periodontitis, and ultimately tooth loss—but this clinical understanding lacks quantitative network-level evidence from large-scale population data. Furthermore, whether comorbidity patterns evolve with age, and whether known sex differences [18,19] are structurally reflected at the network level, remain open questions. Translating the intra-oral comorbidity structure into a computable, age-resolved network would also create a substrate on which downstream engineering applications—clinical decision support, risk-prediction modelling, and screening-pathway optimization—can be built.

The present study aimed to construct and characterize the first intra-oral comorbidity network at ICD-10 four-character granularity, using real-world outpatient data from 583,614 patients spanning 15 years (2011–2025) at a specialized dental hospital (75 disease nodes). Specifically, the study aimed to (i) delineate age-stratified community structures, (ii) test whether the clinically recognized caries–pulpitis–tooth-defect progression chain is quantitatively supported by temporal and quasi-causal evidence, and (iii) examine sex-specific network differences. Methodologically, the framework first delineates age-stratified cross-sectional disease communities, dynamically tracks longitudinal progression trajectories at the patient level, and finally applies a suite of time-respecting causal-inference models to evaluate pathway directionality—a three-step progression that organizes the Section 3 that follows.

## 2. Materials and Methods

### 2.1. Study Design and Data Source

This study comprised two parallel analyses: (i) cross-sectional comorbidity networks were constructed within each age stratum to characterize disease co-occurrence structures, and (ii) disease progression trajectories were mined along patient-level longitudinal visit sequences. The data were obtained from the Hospital Information System (HIS) of a specialized dental hospital in China and spanned January 2011 to March 2025. Patients aged 0–100 years with a valid sex record (1 = male, 2 = female) were included. Data quality control consisted of (i) deduplication of same-patient, same-date, same-department, same-diagnosis records; (ii) exclusion of clinically implausible diagnoses (e.g., periodontitis K05.6 in patients aged 0–5 years); and (iii) exclusion of records with extreme visit frequencies (>100 visits per year). The final cohort comprised 583,614 patients with 2,863,671 outpatient visit records; 57.0% were female, the median age was 29 years, and 17 clinical departments were represented. Reporting followed the STROBE guideline [20]. Helsinki-compliant ethical approval (ZJUSS-IRB-2026-164) and a waiver of informed consent are reported in the Institutional Review Board Statement and Informed Consent Statement of the back matter.

### 2.2. ICD-10 Code Processing and Age Stratification

Original diagnostic codes were standardized to ICD-10 four-character codes (e.g., K07.101 → K07.1) [21]. The included codes covered the entire oral-disease chapter K00–K14 as well as oral-related codes, including S02.5 (tooth fracture), S03.2 (tooth luxation), T81.0 (post-extraction hemorrhage), C00–C06 (oral malignant neoplasms), B37.0 (oral candidiasis), G90.6 (burning mouth syndrome), and L43.9 (lichen planus). A frequency threshold of ≥100 visits was applied, yielding 75 four-character code disease nodes. The complete code list is provided in Table S1.

Based on the clinical characteristics of oral diseases, five unequal-width age strata were used (Table 1): L1 (0–17 years, 124,950 patients; deciduous caries, mixed dentition, early orthodontics), L2 (18–29 years, 187,338 patients; peak orthodontics, third molars, gingivitis), L3 (30–44 years, 172,531 patients; early periodontitis, pulp disease), L4 (45–59 years, 81,960 patients; periodontal progression, onset of tooth loss), and L5 (60+ years, 56,637 patients; tooth loss, prosthetic needs). The same patient could be assigned to different age strata at different visit ages; patients crossing age strata accounted for 6.8%.

### 2.3. Comorbidity Network Construction

Following the operational definition used in large-scale comorbidity network studies [1,4], a comorbidity pair was defined when a patient received diagnoses of two different diseases across any visits within the same age stratum, regardless of whether these visits occurred on the same day or on different days. This definition captures sequential co-occurrence (diseases diagnosed at different time points) rather than strictly concurrent comorbidity; as in prior EHR-based comorbidity studies, disease resolution cannot be reliably determined from administrative data, and the two definitions converge for chronic conditions. Because each visit recorded only one ICD-10 code (a system constraint of the hospital information system), co-occurrence necessarily required at least two separate visits. The same patient could contribute to different age strata if their visits crossed stratum boundaries during the 15-year follow-up; patients with visits in more than one age stratum (*n* = 39,686; 6.8% of all patients) appeared in the comorbidity tabulation of each stratum to which they had visits, but their diagnoses in stratum *L*_k_ were only paired with their other diagnoses in stratum *L*_k_. Within-stratum diagnostic pairs (regardless of the gap between visits, including gaps longer than 30 days) defined the cross-sectional comorbidity edges; for the longitudinal progression-trajectory and TPR analyses, the patient’s full sequence across all strata was preserved (no truncation at stratum boundaries), so that cross-stratum sequences such as a diagnosis at age 17 followed by another at age 18 were retained. For each disease pair, a 2 × 2 contingency table was constructed and the relative risk (RR) was calculated:

(1) $$RR_{ij}=\frac{P(j|i)}{P(j|\neg i)}$$

Although the measure is technically a prevalence ratio (PR), given that it is computed from cross-sectional prevalence within age strata rather than from incidence rates in a prospective cohort, the term “relative risk (RR)” is retained for consistency with the established comorbidity network literature [1,4]. Fisher’s exact test assessed significance, with Bonferroni correction for multiple comparisons [22]. Edge inclusion criteria were RR > 1.5 [1,4,22] (validated across 1.2–3.0 in sensitivity analysis in Supplementary Materials S2), corrected *p* < 0.05, all contingency-table cells ≥ 5 patients, and co-occurring patient count ≥ 10. Edge weights were defined as log_2_(RR). Six undirected weighted comorbidity networks were constructed for the five age strata and the all-age cohort.

Operational definition of comorbidity and framework positioning. Throughout this study, “comorbidity” is used in the broad EHR-network sense—any co-occurrence of two or more diseases in the same patient—consistent with prior large-scale comorbidity network studies [1,4,8]. This operational definition deliberately includes two distinct co-occurrence patterns: (i) classical comorbidities, i.e., distinct pathophysiological entities co-occurring without a direct linear-progression link (such as the oral mucosal immune cluster identified in this study, comprising lichen planus, candidiasis, xerostomia, and burning mouth syndrome), and (ii) quasi-causal progression sequences, i.e., sequential clinical stages of an interrelated disease process (such as caries → pulpitis → tooth defect). The framework distinguishes these two patterns analytically: Louvain community detection (Section 2.5) surfaces clusters of either type, whereas the quasi-causal inference suite (Section 2.6—Cox regression, negative outcome controls, and Baron–Kenny mediation) specifically identifies and quantifies linear-progression chains. Modeling the sequential stages of a progression chain as distinct ICD-10 four-character nodes—rather than collapsing them into a single three-character disease category (e.g., “K02 dental caries”)—provides clinically actionable resolution: in this study, it enabled the identification of pulpitis as the complete mediator between caries and tooth defect, with direct implications for the timing of endodontic intervention (Section 4.3).

Single-code-per-visit constraint and its mitigation. Because each visit records only one ICD-10 code, a comorbidity pair necessarily requires at least two separate visits. This raises the concern that concurrent presentations (e.g., simultaneous caries and pulpitis on day one) might artificially generate a forward temporal sequence (A → B) because the clinician chose only one of the two codes and the second appeared at a subsequent visit. Three pre-specified analyses were used to assess and mitigate this concern: (i) the inter-diagnosis gap distribution (Table S7) shows that the proportion of same-day co-diagnoses is low for pathway pairs (1.6% for caries–pulpitis, 3.3% for pulpitis–tooth defect, 6.1% for periapical–tooth defect), so the bulk of observed comorbidities reflect genuinely separated visits; (ii) gap-stratified temporal precedence ratios (Table S8) remain robust for pathway pairs at progressively higher minimum-gap thresholds (≥30, ≥180, ≥365, and ≥730 days), while negative-control pairs exhibit progressively reversed directionality, confirming that the temporal signal is not an artifact of the single-code constraint; and (iii) visit-frequency tertile stratification (Table S9) further shows that TPR patterns are consistent across low-, mid-, and high-frequency strata.

### 2.4. Network Topology and Centrality Analysis

Global topological metrics included number of nodes, number of edges, density, average degree, clustering coefficient, average path length, modularity, and assortativity [23]. Node-level metrics included degree (reported as raw edge counts), weighted degree, betweenness centrality, PageRank, closeness centrality, and eigenvector centrality. Centrality metrics of ten major oral diseases were tracked across age strata and plotted as age–evolution curves. Three inter-layer network similarity metrics quantified structural similarity between age strata (details in Supplementary Methods SM1).

### 2.5. Community Detection and Disease Progression Trajectory Mining

The Louvain algorithm [24] was used to identify comorbidity communities, with a fixed random seed (seed = 42) to ensure reproducibility [25]. Community detection was run separately for each age stratum, and the age stability of community composition was analyzed.

For each patient, all diseases were ordered by first diagnosis date to construct patient-level longitudinal sequences. Frequent sequence mining was performed to extract 2-sequences and 3-sequences (support ≥ 50 patients, minimum interval ≥ 30 days). The denominator for support percentage was the total number of patients with ≥ 2 different diagnoses (*N* = 370,192; 63.4% of all patients). A directed progression network was constructed based on frequency direction ratios. Bifurcation path analysis was performed for five hub diseases (K02.9 dental caries, K04.0 pulpitis, K04.4 apical periodontitis, K05.6 periodontitis, K08.1 partial edentulism), and convergence source analysis was performed for three terminal diseases (K08.1 partial edentulism, K08.3 retained root, K07.1 malocclusion).

To evaluate temporal consistency of the key progression pathway (caries → pulpitis → tooth defect), three analyses were performed. First, the temporal precedence ratio (TPR) was computed for each disease pair. For a disease pair (A, B), TPR was defined as the proportion of co-occurring patients in whom A was first diagnosed ≥ 30 days before B:

(2) $$TPR_{A\to B}=\frac{n(A\to B)}{n(A\to B)+n(B\to A)}$$

Patients with concurrent diagnoses (<30 days apart) were excluded from the denominator. A TPR significantly > 0.5 (two-sided binomial test) indicates temporal directionality. Second, temporal cumulative incidence was estimated by Kaplan–Meier methods (lifelines 0.30.0) at 3, 6, 12, 24, and 60 months after the first diagnosis of disease A; patients who did not develop disease B were censored at their last recorded visit, with 95% confidence intervals obtained from the Greenwood formula. Because non-informative censoring is assumed, these estimates should be interpreted with caution. Third, for patients with all three diagnoses (caries, pulpitis, tooth defect), the empirical distribution of the six possible temporal orderings was compared against the null expectation of 16.7% per ordering.

### 2.6. Quasi-Causal Inference Analyses

To move beyond temporal precedence toward causal inference, three primary analyses were performed, with three additional sensitivity analyses in the Supplementary Materials. Cox proportional hazards regression assessed each pathway pair, with disease A as the exposure and time-to-disease-B as the outcome, adjusting for baseline age, sex, and log-transformed total visit count (proxy for healthcare utilization); hazard ratios (HR) with 95% confidence intervals were reported. Negative outcome controls evaluated biological specificity: the ability of dental caries to predict diseases outside the caries–pulpitis–periapical chain was assessed in the same Cox framework, with oral candidiasis (B37.0) and lichen planus (L43.9) as negative outcomes; a pathway-specific signal should show HR > 1 for pathway outcomes and HR ≤ 1 for unrelated outcomes. Baron–Kenny mediation analysis [26] with logistic regression was performed on the three-step pathway caries → pulpitis → tooth defect to quantify the proportion of the caries → tooth defect association mediated through pulpitis, adjusting for age, sex, and visit count; 95% confidence intervals for the proportion mediated were obtained from 500 bootstrap resamples. Because this analysis used cross-sectional binary indicators (ever-diagnosed), the results quantify statistical mediation and should be interpreted as supportive rather than definitive causal evidence. Three additional quasi-causal analyses are reported in Supplementary Results S9: inter-diagnosis gap stratification (minimum gap 30–730 days), visit frequency stratification (Berkson’s bias assessment), and constraint-based causal discovery using the Peter–Clark (PC) algorithm and Fast Causal Inference (FCI) algorithm [27] with IDA-based causal effect estimation [28].

Time-varying exposure, immortal time bias, and surveillance bias. Longitudinal EHR data is intrinsically vulnerable to immortal time bias (patients accumulating more follow-up time are more likely to receive subsequent diagnoses) and surveillance bias (patients who remain active in the tertiary system longer are subject to more diagnostic opportunities). The Cox model in this study includes log-transformed total visit count as a time-invariant covariate, which is a first-line adjustment for utilization heterogeneity but does not fully eliminate time-varying surveillance differences. Three supporting analyses address this: (i) the visit-frequency tertile sensitivity analysis (Table S9) shows that key TPR patterns are consistent across low-, mid-, and high-frequency strata, indicating the directional signal is not solely a product of differential surveillance; (ii) negative outcome controls (caries → oral candidiasis, caries → lichen planus) show HR substantially below 1.0, demonstrating that the Cox model is not generically amplifying associations among frequent attenders; and (iii) gap-stratified TPR (Table S8) confirms directional persistence at minimum gaps of one to two years, well beyond the median follow-up time of most short-attending patients. These triangulating sensitivities collectively reduce—though do not eliminate—concerns about time-varying confounding, and residual limitations are discussed in Section 4.5.

**Time-respecting causal-direction analysis on the full comorbidity network.** Because the 15-year EHRs carry visit-level timestamps, a time-respecting causal-direction analysis was performed in parallel with the constraint-based PC/FCI baseline to address the methodological concern that flattening longitudinal data into cross-sectional binary indicators discards the temporal signal. For every edge in the all-age comorbidity network (*n* = 167), the patient-level first-diagnosis dates of the two end-nodes were used to count forward and reverse transitions under a 30-day minimum gap, producing a TPR-based edge orientation that is analogous in scope to PC’s orientation step but explicitly grounded in temporal precedence. Edges with Bonferroni-corrected two-sided binomial *p* < 0.05 were directed; the remaining edges (typically co-occurring or chronic conditions diagnosed within the 30-day window) were left undirected. The resulting time-respecting directed acyclic graph (T-DAG) was compared head-to-head with the PC-derived DAG to quantify the degree of mis-orientation produced by PC when restricted to cross-sectional binary inputs.

### 2.7. Sex-Stratified Comparison and Sensitivity Analysis

Sex-stratified all-age comorbidity networks were constructed separately for male and female patients, and topology, centrality rankings, community structures, and sex-specific edges were compared. The robustness of the conclusions was systematically tested across eight dimensions: (1) ICD granularity (3- vs. 4-character codes); (2) RR threshold (1.2/1.5/2.0/3.0); (3) age grouping (5 unequal-width strata vs. 8 equal-width 10-year strata); (4) co-occurrence measure (RR vs. Phi coefficient); (5) community algorithm (Louvain vs. Greedy Modularity vs. Label Propagation); (6) time window (30/90/180 days); (7) single-diagnosis bias estimation; and (8) temporal stability (network topology across three 5-year periods). All analyses were performed in Python 3.9.6 (Python Software Foundation, Wilmington, DE, USA), with NetworkX 3.2.1, SciPy 1.13.1, lifelines 0.30.0, statsmodels 0.14.6, and matplotlib 3.9.4. Additional software for supplementary analyses (causal-learn 0.1.4, scikit-learn 1.6.1) is documented in Supplementary Methods SM3.

## 3. Results

### 3.1. Network Overview and Age-Stratified Topology

**Figure 1:**
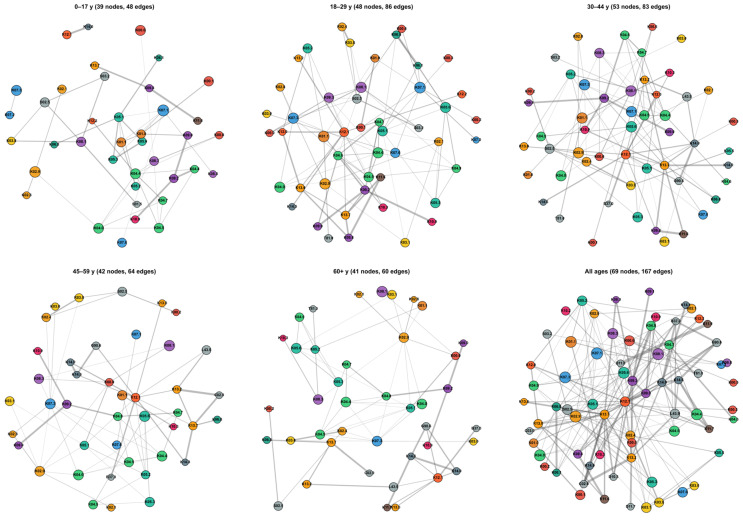
Visualization of age-stratified comorbidity networks (2 × 3 grid: five age strata + all-age network). Node colour indicates the ICD-10 three-character disease category; node size is proportional to log_2_(visit frequency); edge width reflects log_2_(RR). Node labels are ICD-10 four-character codes; full disease names corresponding to each code are listed in Table S1.

The all-age comorbidity network comprised 75 nodes and 167 edges, with density 0.060, average degree 4.45, clustering coefficient 0.414, average path length 3.06, and modularity 0.53 (Figure 1). Patient-level bootstrap resampling (200 iterations) yielded narrow 95% confidence intervals for all metrics (edges [163, 183]; modularity [0.49, 0.57]; see Table 2 footnote), confirming the stability of the observed network structure. Topological metrics of each age-stratum network showed distinct age-dependent changes (Table 2; bar-chart comparisons in Figure S1).

Node counts include all disease codes meeting the frequency threshold (≥100 visits) in each age stratum, including isolated nodes without significant comorbidity edges. The all-age network contained 69 connected nodes and 6 isolated nodes (G50.0, K05.0, K07.0, K11.2, K11.5, S01.5). Bootstrap 95% CIs were obtained by resampling patients with replacement (200 iterations); in each iteration, the entire network was reconstructed. All-age network: edges 167 [163, 183]; density 0.060 [0.059, 0.066]; average degree 4.45 [4.35, 4.88]; clustering coefficient 0.414 [0.356, 0.449]; average path length 3.06 [2.91, 3.25]; and modularity 0.53 [0.49, 0.57]. Per-layer bootstrap CIs are provided in Table S2.

Network complexity (number of edges) peaked in the 18–29 stratum (86 edges), was similar in 30–44 (83 edges), and declined with age to 60 edges in the 60+ stratum. Modularity was highest in 0–17 (0.73), indicating that childhood comorbidities were the most modular and most clearly delimited. Average path length reached its maximum in L1 (4.48) and its minimum in L5 (3.27), showing that the elderly network—although sparser—has a more compact core. Inter-layer similarity analysis confirmed that the L4–L5 transition was the most stable (DeltaCon similarity 0.261), while L1–L2 was the most dramatic (DeltaCon similarity 0.187) (Table S3, Figure S6).

### 3.2. Hub Diseases and Centrality Evolution

**Figure 2:**
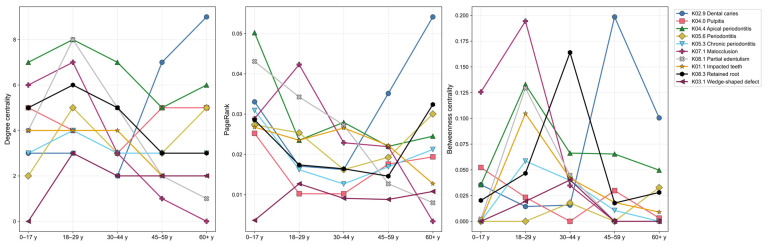
Evolution curves of centrality metrics for major oral diseases across age strata. left: degree centrality; middle: PageRank; right: betweenness centrality.

The centrality metrics of major oral diseases exhibited characteristic evolution curves across age strata (Figure 2).

The degree centrality of dental caries (K02.9) remained low (2–3) from L1 to L3, rose sharply to 7 in L4, and peaked at 9 in L5—the highest value in the entire network; PageRank also peaked in L5 (0.054, Table S2), confirming that dental caries is the strongest comorbidity hub in the 60+ stratum. Apical periodontitis (K04.4) peaked at L1–L2 (degree 7–8) and then declined, suggesting that young adulthood is the stage of most complex periapical comorbidity. Malocclusion (K07.1) dropped from 7 in L2 to 1 in L4 and 0 in L5, indicating that orthodontic-related comorbidities are largely confined to younger populations. Periodontitis (K05.6) maintained degree = 5 in both L2 and L5, demonstrating sustained hub status across age groups—consistent with the 2018 periodontitis staging framework, which recognizes periodontitis as a chronic condition requiring lifelong management [29]. In the all-age network, the nodes with the highest degree centrality were recurrent aphthous ulcer (K12.1, degree = 19) and oral mass (K13.7, degree = 16); gingivitis (K05.1) had the highest betweenness centrality (0.238), serving as a bridging connector between communities.

### 3.3. Disease Community Structure and Age Stability

The Louvain algorithm partitioned the all-age network into eight comorbidity communities (Table S4, Figure S2): a restorative/defect cluster (18 members; the largest, spanning pulpitis through partial edentulism), a developmental/caries cluster (11 members), a cyst/mass cluster (9 members), an extraction/surgical cluster (8 members), an oral mucosal immune cluster (8 members; burning mouth syndrome [global prevalence ≈ 1.7% [30]], lichen planus, candidiasis, xerostomia), a periodontal cluster (7 members), an oral neoplasm cluster (6 members), and a parotid cluster (2 members). Community stability analysis (Figure S3) showed that the periodontal cluster was the most stable across all age strata (normalized mutual information [NMI] between adjacent strata: range 0.72–0.89), while dental caries (K02.9) had the most unstable community assignment—shifting from an independent caries community in L1 to the restorative/defect community in L4–L5 (NMI 0.41–0.58)—reflecting a fundamental shift in the comorbidity pattern of caries with age.

### 3.4. Disease Progression Trajectories

Frequent sequence mining identified 627 two-step sequences and 20 three-step sequences with support ≥ 50. Table 3 lists the five most frequent two-step sequences alongside negative controls.

The full top-10 list is provided in Table S5. Classic textbook pathways were quantitatively characterized: caries → pulpitis → tooth defect was observed in 1846 patients (0.50%); caries → apical periodontitis → tooth defect in 1297 (0.35%); and the periodontitis → partial edentulism degradation chain in 10,357 (2.80%). Bifurcation path analysis (Figure S4) showed that after pulpitis (K04.0), 29.9% of patients progressed to tooth defect (restoration), 16.2% to dental caries (new caries), and 11.5% to apical periodontitis (worsening). After dental caries (K02.9), 14.1% progressed to tooth defect, 12.8% to pulpitis, and 10.9% to apical periodontitis. Convergence source analysis demonstrated that partial edentulism (K08.1) receives upstream flows from at least five distinct pathways—periodontitis (7.9%), tooth defect (6.2%), retained root (5.9%), apical periodontitis (4.8%), and dental caries (4.6%)—highlighting that tooth-loss prevention must simultaneously address multiple upstream conditions.

Temporal consistency analysis provided further evidence for directionality (Table 4). Pulpitis → tooth defect showed the strongest temporal directionality (TPR = 0.771, *p* < 0.001); apical periodontitis → tooth defect was also strongly directional (TPR = 0.680, *p* < 0.001). For caries → pulpitis, the overall TPR was 0.540 (*p* < 0.001), only marginally above chance, reflecting a clinical scenario in which patients often present with symptomatic pulpitis before a separate caries code is recorded; in the pediatric stratum (0–17 years), however, the TPR rose to 0.631 (*p* < 0.001). Negative-control pairs showed reversed or near-random temporal directionality (TMJ disorder → pulpitis: TPR = 0.369; malocclusion → pulpitis: TPR = 0.459), confirming pathway specificity. The full temporal precedence analysis is provided in Table S6.

Kaplan–Meier temporal cumulative incidence analysis showed monotonically increasing cumulative incidence for all pathway pairs. The cumulative incidence of tooth defect following pulpitis was 32.9% (95% CI 32.5–33.3%) at 3 months and 57.2% (56.6–57.8%) at 5 years, while the negative control pair (TMJ disorder → pulpitis) showed minimal incidence (1.2% at 3 months, 13.2% at 5 years). Among 2240 patients with all three diagnoses, the ordering caries → pulpitis → tooth defect was the most frequent (951 patients, 42.5%), 2.5-fold enriched over the null expectation of 16.7% (χ^2^ test, *p* < 0.001).

Analysis of inter-diagnosis gap distributions revealed that the proportion of same-day co-diagnoses was low for pathway pairs (caries–pulpitis: 1.6%; pulpitis–tooth defect: 3.3%), and the majority of co-occurring patients had gaps exceeding 30 days, reducing concern that the single-code-per-visit constraint artificially generated the observed temporal patterns (Table S7).

### 3.5. Quasi-Causal Inference Analyses: Cox Regression, Negative Controls, and Mediation

Three quasi-causal analyses supported the directionality and specificity of key progression pathways (Table 5).

Cox regression. After adjusting for age, sex, and total visit count (proxy for healthcare utilization), HRs remained large and significant for all pathway pairs, while negative exposure controls yielded HR < 1 (TMJ → pulpitis: HR = 0.399; malocclusion → pulpitis: HR = 0.162), indicating that the associations are not driven by healthcare utilization confounding.

Negative outcome controls. Caries predicted pathway outcomes (HR > 1) but did not predict clinically unrelated diseases (oral candidiasis: HR = 0.145; lichen planus: HR = 0.409; both with HR well below 1.0), confirming biological specificity.

Mediation analysis. Baron–Kenny analysis showed that pulpitis completely mediates the caries → tooth defect association: the total effect was modest (OR = 1.082, *p* < 0.001), the direct effect was null (OR = 0.996, *p* = 0.60), and the indirect effect through pulpitis was strong (OR = 2.282, *p* < 0.001). The proportion mediated was approximately 100% (point estimate 105%, 95% CI 90–128%), consistent with complete mediation. The proportion mediated through periapical periodontitis was 12.2%.

**Figure 3:**
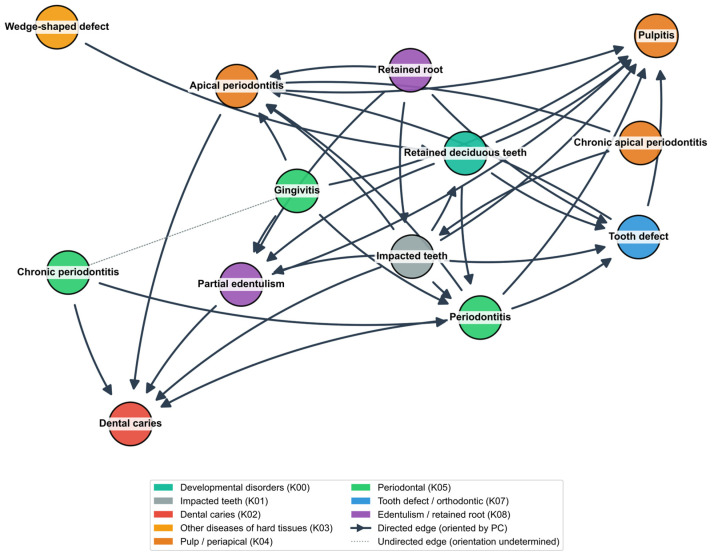
PC algorithm DAG for 13 key oral diseases. The PC-derived DAG reversed the direction of caries and pulpitis, placing them as terminal sinks, demonstrating that constraint-based DAG learning on cross-sectional binary data identifies conditioning structure rather than temporal causation. Solid arrows indicate directed edges (oriented by PC); dotted lines indicate undirected edges (orientation undetermined).

Three additional quasi-causal sensitivity analyses—gap-stratified TPR, visit frequency stratification, and PC/FCI causal discovery with IDA estimation—are reported in Supplementary Results S9 and yielded consistent results. The PC algorithm, when applied to cross-sectional binary indicators, reversed the topological order of caries and pulpitis (placing both as terminal sinks), demonstrating that constraint-based DAG learning on cross-sectional binary data identifies statistical conditioning structure rather than temporal causation (Figure 3).

To directly address the methodological criticism that flattening longitudinal records into cross-sectional inputs discards temporal signal, a time-respecting causal-direction analysis (T-DAG) was performed on the full all-age comorbidity network (167 edges). Of these, 45 edges (26.9%) carried a Bonferroni-significant temporal direction based on patient-level first-diagnosis precedence; the remaining 122 edges either co-occurred within the 30-day concurrency window or lacked enough directional cases to reach significance (typically chronic conditions diagnosed in parallel). All key clinical pathway edges retained in the all-age network were temporally oriented in the clinically expected direction: pulpitis → tooth defect (TPR = 0.771, *p*_Bonf_ < 0.001), apical periodontitis → tooth defect (TPR = 0.680, *p*_Bonf_ < 0.001), and periodontitis → partial edentulism (TPR = 0.636, *p*_Bonf_ < 0.001). Head-to-head comparison with the PC-derived DAG (full edge-level results in Table S10) found that, among the 8 edges where both methods returned a direction, the PC algorithm reversed the clinically expected (and time-respecting) orientation in 5 of 8 cases (62.5%). This empirically confirms that the cross-sectional PC algorithm is unsuited as a primary causal-discovery tool for longitudinal EHR data; PC was included here as a methodological baseline to expose this limitation, while the time-respecting analyses—TPR, Cox regression with temporally ordered exposure, Baron–Kenny mediation, and the full-network T-DAG—constitute the primary causal evidence.

### 3.6. Sex Differences

**Figure 4:**
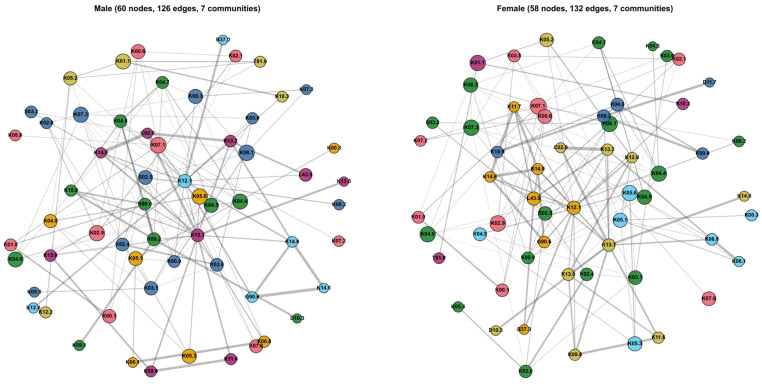
Sex-stratified comorbidity network comparison. (**Left**): male network; (**right**): female network. Sex-specific edges are highlighted. Node colours indicate Louvain community assignment; node labels are ICD-10 four-character codes (full disease names are listed in Table S1). Node colour denotes Louvain community membership (resolution = 1.0, seed = 42). Edge line width is proportional to log_2_(RR) of the comorbidity pair, identical to the encoding used in Figure 1.

The all-age comorbidity networks for males and females showed significant topological differences (Figure 4 and Figure S7). The male network had 126 edges with 27 male-specific edges; the female network had 132 edges with 33 female-specific edges and stronger local clustering (clustering coefficient 0.342 vs. 0.290). Sex-specific comorbidity associations had clear clinical implications (Table 6).

Male-specific edges predominantly involved oral neoplasms and precancerous lesions, reflecting clustering driven by male risk factors such as smoking and alcohol [31]. Female-specific edges predominantly involved xerostomia and immune-mediated mucosal diseases, likely related to the female predominance of Sjögren’s syndrome (female-to-male ratio 9–10:1) [32,33].

### 3.7. Sensitivity Analysis

The eight-dimensional sensitivity analysis systematically tested the robustness of the main findings (Table 7).

When the RR threshold varied from 1.2 to 3.0, the number of edges decreased from 191 to 106, but hub node rankings remained stable. The three community detection algorithms produced highly consistent results. The single-diagnosis bias analysis (S7) confirmed that only 7 edges survived when co-occurrence was restricted to strict same-day encounters—compared with 167 in the main network—demonstrating that the cross-visit design captures a substantially broader comorbidity structure. The temporal stability analysis (S8) found 21 edges stable across all three periods, including pathologically fundamental associations such as periapical periodontitis ↔ periapical abscess and recurrent aphthous ulcer ↔ lichen planus. Detailed sensitivity panels are provided in Figure S5.

## 4. Discussion

### 4.1. Major Findings and Network-Medicine Implications

This study constructed a comprehensive comorbidity network within oral diseases based on ICD-10 four-character codes, addressing the gap in the literature where intra-oral comorbidity relationships have not been systematically described at this granularity. Unlike prior studies [9,10] that focused on oral–systemic comorbidities, this study maps the compartmentalized topological architecture of the intra-oral disease spectrum using 75 four-character code nodes. The eight communities in the all-age network included both clinically expected clusters—such as the periodontal cluster (K05.x) and the restorative/defect cluster (K04 → K07.3 → K08)—and findings beyond expectations: the oral mucosal immune cluster formed a separate community independent of the oral neoplasm cluster. This cluster—comprising burning mouth syndrome (G90.6), lichen planus (L43.9), oral candidiasis (B37.0), and xerostomia (K11.7)—suggests that these immune-mediated and dryness-related mucosal diseases may share pathogenic mechanisms, possibly related to salivary hypofunction or autoimmune dysregulation. This interpretation is supported by evidence that oral lichen planus (OLP) is associated with autoimmune thyroid disease and diabetes [34] and with multiple systemic conditions and medications [35], and by multi-center data showing that Chinese OLP patients frequently present with comorbid mucosal complaints [36]. Clinically, this finding supports a combined screening approach: when a patient presents with one of these conditions (e.g., lichen planus), clinicians should actively assess for the others (xerostomia, candidiasis), particularly in middle-aged and elderly women where these conditions cluster most strongly.

Methodologically, this study aligns with the multilayer age-stratified framework of prior comorbidity-network work [4] but differs in two key respects: (i) it operates at the subspecialty level—75 four-character codes within oral diseases—rather than at the cross-specialty level of ICD chapters or three-character codes, and (ii) it uses outpatient data from a specialized dental hospital rather than inpatient data from a general hospital, capturing earlier disease stages and ambulatory-care patterns that are invisible in hospitalization-based comorbidity networks. From a network-medicine perspective, these findings corroborate the principle [3] that diseases with overlapping molecular neighborhoods tend to co-occur. While this study operates at the phenotypic rather than the molecular level, the formation of clinically coherent disease communities suggests that shared pathobiological mechanisms drive comorbidity clustering even within a single clinical specialty. From a bioengineering perspective, the age-resolved network is itself a reusable computational substrate on which predictive analytics and decision-support tools can be built.

### 4.2. Clinical Implications of Age–Evolution Patterns

Network complexity peaked in the 18–29 stratum because multiple diseases—orthodontic conditions, third molars, caries, and initial periodontitis—intersect during this period. The high visit frequency of orthodontic patients (monthly recalls over 2–3 years) may also contribute to L2 complexity by increasing opportunities for co-diagnosis; malocclusion (K07.1) reached degree = 7 in L2, although this reflects genuine clinical co-management rather than a purely artifactual signal. The degree centrality of dental caries (K02.9) reached 9 in the 60+ stratum—the highest value in the network—quantitatively confirming the clinically recognized comorbidity burden of caries in older adults: caries in this group is not merely an isolated condition but forms a complex comorbidity network with periodontal disease, prosthetic needs, and tooth loss. This network analysis adds a structural dimension to existing evidence on the oral-disease burden in older adults [37,38]: as the highest-degree hub, dental caries connects periodontal disease, tooth loss, and prosthetic needs into a tightly coupled module, suggesting that interventions targeting caries in elderly patients could yield cascading benefits across the cluster. Periodontitis maintained degree = 5 across both young adulthood and old age, demonstrating sustained hub status consistent with its chronic progressive nature [29,39], whereas malocclusion fully detached from the network after age 45, indicating a strict age window for orthodontic-related comorbidities. These findings translate into specific clinical recommendations: for older adults (60+), each dental visit should include concurrent caries and periodontal screening, and early caries intervention in this group may yield broad cascading benefits across the comorbidity cluster; for young adults (18–29), clinicians should be alert to periapical complications during orthodontic treatment, given the peak comorbidity complexity in this stratum.

### 4.3. Quasi-Causal Evidence for Progression Pathways

The quasi-causal analyses strengthened the observational findings beyond simple temporal precedence.

Cox regression adjusted for age, sex, and healthcare utilization confirmed that the pathway associations are not driven by differential healthcare-seeking behavior. After adjustment, pathway pairs retained large hazard ratios (pulpitis → tooth defect: HR = 2.65; caries → pulpitis: HR = 2.25), while negative controls showed no positive association (HR < 1). Although the cross-sectional direct association between caries and tooth defect was modest (RR = 1.13; Cox HR = 1.04), the mediation analysis proved informative on precisely this point: the caries → tooth defect path operates almost entirely through pulpitis rather than as a direct effect. Negative outcome controls further demonstrated that caries predicts pathway diseases but not clinically unrelated diseases (oral candidiasis: HR = 0.15; lichen planus: HR = 0.41), confirming biological specificity rather than a generic healthcare-utilization effect. Additional robustness checks—visit-frequency stratification (Table S9) and gap-stratified TPR (Table S8)—further supported these findings.

Building on this, the mediation analysis showed that pulpitis completely mediates the caries → tooth defect association (proportion mediated ≈ 100%, 95% CI 90–128%), with no residual direct effect (OR = 0.996, *p* = 0.60). This quantitative demonstration that caries does not independently lead to tooth defect but rather acts through pulpitis and provides population-scale support for the clinical consensus that timely pulpitis management is critical for preventing tooth-structure loss. Clinically, this implies that the window between caries progression to pulp involvement and subsequent structural damage represents the most effective intervention point: early endodontic treatment or vital pulp therapy at this stage may prevent the downstream cascade toward tooth defect and eventual tooth loss.

The constraint-based PC algorithm (Figure 3) was included as a methodological baseline—not as a primary causal estimator—to empirically demonstrate why flattening longitudinal EHRs into cross-sectional binary inputs is inadequate for causal discovery in this domain. The PC-derived DAG reversed the direction of several clinically established pathways and placed caries and pulpitis as terminal sinks, whereas the head-to-head time-respecting T-DAG analysis on the full 167-edge network (Section 3.5) recovered the clinically expected orientations and revealed that PC reversed direction in 5 of 8 overlapping edges (62.5%). The convergence of TPR, Cox regression, mediation, and the T-DAG—all of which exploit timestamps—versus the divergence of cross-sectional PC illustrates that the limitation lies in the input representation (cross-sectional binary indicators), not in the constraint-based algorithm itself [40]. This is consistent with the broader principle that, for longitudinal EHR data, time-respecting methods are necessary for valid causal inference.

Although these analyses do not constitute formal causal proof, the consistent results across confounding adjustment, specificity testing, mediation decomposition, and additional sensitivity analyses (Supplementary Results S9) provide stronger support for disease progression directionality than temporal precedence alone.

### 4.4. Sex Differences: Biological Interpretation

Male and female comorbidity networks exhibited structural differences reflecting known epidemiological patterns: male-specific edges involved oral neoplasm co-occurrence driven by smoking and alcohol risk factors [31], while female-specific edges involved xerostomia and immune-mediated mucosal diseases related to the female predominance of Sjögren’s syndrome [32]. The department transfer network further corroborated disease progression patterns: the Department of Operative Dentistry and Endodontics was the largest source and the Department of Oral and Maxillofacial Surgery the largest sink (Supplementary Results S9.4).

### 4.5. Limitations

The present findings should be interpreted in light of the following limitations.

Single-center referral bias (Berkson’s bias). Data were drawn from a single specialized tertiary dental hospital. Patients attending such a center are pre-selected for higher disease severity, more advanced symptoms, and greater healthcare-utilization density than the general population, which biases the observed RR magnitudes and network density upward—a classical form of Berkson’s referral bias [40]. Although the converging sensitivity analyses (visit-frequency stratification, gap-stratified TPR, and the constraint-based DAG comparison) all support the directional architecture of the network, the absolute magnitudes of edge weights and the prevalence of less-common nodes should not be extrapolated to community-based populations without external validation. Multi-center replication that includes primary-care and community-clinic data is needed to establish population-level transportability.

Single-code-per-visit constraint. The hospital information system records only one ICD-10 code per visit, so any comorbidity pair requires at least two separate visits. While Section 2.3 reports three sensitivity analyses that show the single-code constraint does not artificially generate the observed directionality (low same-day co-diagnosis fractions, gap-stratified TPR robustness, and frequency-tertile consistency), the constraint still attenuates absolute estimates of concurrent comorbidity and prevents direct quantification of simultaneous-presentation cases.

ICD granularity. ICD-10 four-character codes cannot capture the 2018 periodontitis staging/grading system [29]; K05.3 (chronic periodontitis, former classification) and K05.6 (periodontitis, current classification) form a strong edge (RR = 4.85) that reflects coding redundancy rather than true comorbidity.

Time-varying surveillance and residual confounding. This remains an observational study. The Cox models adjust for log-transformed visit count but cannot fully eliminate immortal time bias or time-varying surveillance bias (weaker associations such as caries → tooth defect HR = 1.04 should therefore be interpreted with caution). The mediation analysis used ever-diagnosed cross-sectional indicators rather than time-ordered variables; detection bias most likely explains the weak directionality for the caries → pulpitis pair, where symptomatic pulpitis often prompts the visit at which caries is incidentally recorded.

No systemic-disease or tooth-level linkage. No systemic disease or tooth-level data were available; future studies incorporating these data would enable oral–systemic interaction analysis and within-tooth progression tracking.

## 5. Conclusions

This study mapped an intra-oral comorbidity network at ICD-10 four-character resolution using data from 583,614 patients. The network revealed eight age-evolving disease communities, in which dental caries emerged as the dominant hub in older adults (degree = 9) and periodontitis maintained sustained importance across age groups. Cox regression and mediation analysis provided quasi-causal support for key progression pathways; in particular, pulpitis was shown to completely mediate the caries-to-tooth-defect association, identifying early pulpitis management as the critical intervention point. Sex-stratified networks reflected distinct risk profiles, and the main findings remained robust across multiple sensitivity dimensions. From a bioengineering perspective, the reproducible age-stratified network-mining pipeline provides a substrate for the development of age- and sex-specific risk-stratified oral-disease management and downstream clinical decision-support systems.

## Figures and Tables

**Table 1:** Age stratification and patient distribution.

Age Stratum	Age Range	No. of Patients	Oral Disease Characteristics
L1	0–17 years	124,950	Deciduous caries, mixed dentition, early orthodontics
L2	18–29 years	187,338	Peak orthodontics, third molars, gingivitis
L3	30–44 years	172,531	Early periodontitis, pulp disease
L4	45–59 years	81,960	Periodontal progression, onset of tooth loss
L5	60+ years	56,637	Tooth loss, prosthetic needs

**Table 2:** Topological metrics of comorbidity networks by age stratum.

Age Stratum	Nodes	Edges	Density	Average Degree	Clustering Coefficient	Average Path Length	Modularity
L1 (0–17)	54	48	0.034	1.78	0.202	4.48	0.73
L2 (18–29)	65	86	0.041	2.65	0.284	3.91	0.68
L3 (30–44)	71	83	0.033	2.34	0.192	3.77	0.57
L4 (45–59)	66	64	0.030	1.94	0.259	3.83	0.61
L5 (60+)	63	60	0.031	1.90	0.191	3.27	0.71
All ages	75	167	0.060	4.45	0.414	3.06	0.53

**Table 3:** Top 5 most frequent disease progression sequences and negative controls.

Rank	Sequence	No. of Patients	Support (%)	RR (95% CI)
1	Dental caries → Tooth defect	16,934	4.58	1.13 (1.12–1.14)
2	Pulpitis → Tooth defect	16,429	4.44	2.11 (2.08–2.13)
3	Apical periodontitis → Tooth defect	12,708	3.43	1.66 (1.64–1.68)
4	Dental caries → Pulpitis	11,961	3.23	1.47 (1.45–1.49)
5	Dental caries → Apical periodontitis	11,472	3.10	1.08 (1.06–1.09)

**Table 4:** Temporal precedence analysis of key disease pairs.

Disease Pair	TPR	Binomial *p*	Median Gap (Days)	Type
Pulpitis → Tooth defect	0.771	<0.001	77	Pathway
Periapical → Tooth defect	0.680	<0.001	135	Pathway
Caries → Tooth defect	0.601	<0.001	283	Pathway
Caries → Pulpitis	0.540	<0.001	247	Pathway
Malocclusion → Pulpitis	0.459	<0.001	286	Negative ctrl
TMJ disorder → Pulpitis	0.369	<0.001	304	Negative ctrl

Patients with concurrent diagnoses (<30 days apart) were excluded from the TPR denominator. Median gap was computed among forward cases only.

**Table 5:** Quasi-causal inference results for key progression pathways.

Disease Pair	Cox HR (95% CI) ^a^	Negative Outcome HR ^b^	Type
Pulpitis → Tooth defect	2.652 (2.613–2.691) ***	—	Pathway
Caries → Pulpitis	2.248 (2.207–2.290) ***	—	Pathway
Periapical → Tooth defect	1.642 (1.617–1.668) ***	—	Pathway
Caries → Periapical	1.308 (1.284–1.332) ***	—	Pathway
Caries → Candidiasis	—	0.145 (0.052–0.404) ***	Negative ctrl
Caries → Lichen planus	—	0.409 (0.332–0.504) ***	Negative ctrl
TMJ → Pulpitis	0.399 (0.362–0.439) ***	—	Negative ctrl

^a^ Cox proportional hazards model adjusted for age, sex, and log(visit count). ^b^ HR for caries predicting clinically unrelated diseases. *** *p* < 0.001.

**Table 6:** Sex-specific comorbidity associations.

Sex	Comorbidity Association	RR (95% CI)	Co-Occurring Cases	Clinical Interpretation
Male	Tongue mass ↔ Malignant neoplasm of tongue	419.4 (217.5–808.6)	10	Extremely strong oral neoplasm co-occurrence in males
Male	Oral mass ↔ Oral mucosal hyperplasia	42.9 (22.8–80.6)	12	Proliferative oral mucosal lesions
Male	Jaw cyst ↔ Nasopalatine duct cyst	41.0 (25.6–65.7)	22	Jaw cystic disease clustering
Male	Oral leukoplakia ↔ Glossitis	23.8 (13.1–43.4)	11	Precancerous lesion co-occurrence
Female	Glossodynia ↔ Xerostomia	162.3 (101.3–259.9)	18	Oral dryness syndrome-related
Female	Glossitis ↔ Xerostomia	77.9 (46.3–131.1)	15	Oral dryness/immune-mediated
Female	Oral mass ↔ Benign oral neoplasm	44.9 (23.7–85.1)	11	Benign oral tumors
Female	Cheilitis ↔ Herpetic gingivostomatitis	19.2 (11.0–33.4)	13	Oral mucosal inflammation co-occurrence

**Table 7:** Summary of sensitivity analyses.

Dimension	Parameter Variation	Impact on Core Findings	Robustness
S1 ICD granularity	3-character (28 nodes/45 edges) vs. 4-character (75 nodes/167 edges)	Four-character codes provide richer structure; community directions consistent	Robust
S2 RR threshold	1.2/1.5/2.0/3.0 → 191/167/135/106 edges	Hub node rankings stable	Robust
S3 Age grouping	5 unequal-width vs. 8 equal-width 10-year strata	Topological trends consistent	Robust
S4 Co-occurrence measure	RR (167 edges) vs. Phi > 0.02 (101 edges)	Core edges highly overlapping	Robust
S5 Community algorithm	Louvain (Q = 0.584 *)/Greedy (0.531)/LabelProp (0.532)	Major communities highly consistent	Robust
S6 Time window	30 d/90 d/180 d → 627/577/509 frequent 2-sequences	Top sequence rankings stable	Robust
S7 Single-diagnosis bias	Same-day multi-visit: 4.56% of patient-days; same-day-only RR-filtered: 7 vs. 167 main	Cross-visit design captures broader comorbidity structure; main estimates conservative	Informative
S8 Temporal stability	3 periods (2011–15/2016–20/2021–25): 21 stable edges; top-10 sequences consistent	Core structure stable; early-period smaller samples limit power	Moderate

* S5 modularity was calculated on the 69-node subgraph with edges; the all-age modularity 0.53 in Table 2 was based on all 75 nodes (including 6 isolated nodes).

## Data Availability

All analysis code and aggregated results (network edges, centrality metrics, community assignments, sensitivity analyses, and quasi-causal analysis outputs) are publicly available at https://github.com/duzida/oral-comorbidity-network (accessed on 31 May 2026). Raw patient-level data cannot be shared due to privacy constraints but are available from the corresponding author upon reasonable request, subject to institutional data governance approval.

## Supplementary Materials

The following supporting information can be downloaded at: https://www.mdpi.com/article/10.3390/bioengineering13070761/s1, Table S1: The 75 ICD-10 four-character disease codes included in the analysis; Table S2: Bootstrap 95% confidence intervals for network topology metrics; Table S3: Inter-layer network similarity metrics; Table S4: Disease community membership in the all-age network; Table S5: Top 10 most frequent two-step progression sequences; Table S6: Full temporal precedence analysis; Table S7: Inter-diagnosis gap distribution for key pathway pairs; Table S8: Gap-stratified temporal precedence ratio; Table S9: TPR stratified by visit frequency tertiles; Table S10: Time-respecting T-DAG edges for all 167 all-age network edges and head-to-head comparison with the PC-derived DAG (revised version, new in this revision); Figure S1: Topology bar charts across age strata; Figure S2: Community structure visualization; Figure S3: Community stability heatmap; Figure S4: Bifurcation path analysis for hub diseases; Figure S5: Sensitivity analysis panels; Figure S6: Inter-layer similarity analysis; Figure S7: Sex-stratified network comparison; Supplementary Methods SM1: Inter-layer network similarity metrics; SM2: Temporal precedence ratio (TPR); SM3: Software environment for supplementary analyses; Supplementary Results S9: Quasi-causal sensitivity analyses.
