# Supplementary material for: A Network-Medicine Framework for Intra-Oral Comorbidity: Age-Stratified Clustering and Quasi-Causal Progression Modeling from Outpatient Electronic Health Records
Source: Bioengineering (Basel). 2026 Jun 29;13(7):761. doi: 10.3390/bioengineering13070761 (PMC13404942; doi:10.3390/bioengineering13070761)
Supplement: Supplementary file 1 [file bioengineering-13-00761-s001.zip › bioengineering-4381603 supplementary.pdf]

Supplementary Materials

A Network-Medicine Framework for Intra-Oral Comorbidity: Age-Stratified Clustering and Quasi-Causal Progression Modeling from Outpatient Electronic Health Records

Wei Chen, Peng Huang, Zijian Cheng, Yaowu Chen, Xiang Tian, Yumeng Song, Xiaoyan Chen, Qianming Chen, Rui Zhang *

Submitted to *Bioengineering* (MDPI)

Table of Contents

- Supplementary Methods
  - SM1. Inter-layer network similarity metrics
  - SM2. Temporal precedence ratio (TPR)
  - SM3. Software environment for supplementary analyses
- Supplementary Tables
  - Table S1. The 75 ICD-10 four-character disease codes included in the analysis
  - Table S2. Bootstrap 95% confidence intervals for network topology metrics
  - Table S3. Inter-layer network similarity metrics
  - Table S4. Disease community membership in the all-age network
  - Table S5. Top 10 most frequent two-step progression sequences
  - Table S6. Full temporal precedence analysis
  - Table S7. Inter-diagnosis gap distribution for key pathway pairs
  - Table S8. Gap-stratified temporal precedence ratio
  - Table S9. TPR stratified by visit frequency tertiles
  - Table S10. Time-respecting T-DAG edges for all 167 all-age network edges and head-to-head comparison with the PC-derived DAG
- Supplementary Figures
  - Figure S1. Topology bar charts across age strata
  - Figure S2. Community structure visualization
  - Figure S3. Community stability heatmap
  - Figure S4. Bifurcation path analysis for hub diseases
  - Figure S5. Sensitivity analysis panels
  - Figure S6. Inter-layer similarity analysis
  - Figure S7. Sex-stratified network comparison
- Supplementary Results
  - S9. Quasi-causal sensitivity analyses

Supplementary Methods

SM1. Inter-layer network similarity metrics

Three complementary metrics were used to quantify structural similarity between age-stratum networks:

1. **DeltaCon similarity**: computes node-to-node diffusion affinity matrices and measures the difference in signal propagation patterns via Matusita distance.
2. **Ipsen–Mikhailov spectral distance** : constructs spectral density functions from Laplacian eigenvalues and measures global topological differences via Hellinger distance.
3. **Degree-distribution Jensen–Shannon divergence**: an information-theoretic distance between degree distributions.

SM2. Temporal precedence ratio (TPR)

For each disease pair (A, B), TPR = n_forward / (n_forward + n_reverse), where n_forward is the number of patients whose first diagnosis of A preceded B by ≥ 30 days, and n_reverse is the converse. A two-sided binomial test evaluated whether TPR differed from 0.50. Negative control pairs with no expected causal relationship were tested in parallel.

SM3. Software environment for supplementary analyses

Primary analyses used Python 3.9.6 with NetworkX 3.2.1, SciPy 1.13.1, lifelines 0.30.0, statsmodels 0.14.6, and matplotlib 3.9.4. Supplementary causal discovery used causal-learn 0.1.4 (PC and FCI implementations with Fisher-Z conditional independence test, α = 0.001) and scikit-learn 1.6.1.

Supplementary Tables

Table S1. The 75 ICD-10 four-character disease codes included in the analysis

ICD-10 code	Disease name	Visit frequency	No. of patients	Prevalence
K02.9	Dental caries	311,139	174,119	29.83%
K07.3	Tooth defect	263,924	121,272	20.78%
K01.1	Impacted teeth	226,968	115,800	19.84%
K05.6	Periodontitis	255,446	113,609	19.47%
K08.1	Partial edentulism	371,715	101,785	17.44%
K07.1	Malocclusion	585,945	100,972	17.30%
K04.4	Apical periodontitis	199,460	95,521	16.37%
K04.0	Pulpitis	198,407	95,402	16.35%
K08.3	Retained root	55,590	38,092	6.53%
K05.3	Chronic periodontitis	50,207	34,799	5.96%
K05.1	Gingivitis	39,609	31,868	5.46%
K04.5	Chronic apical periodontitis	51,487	27,958	4.79%

The full 75-code list is available from the corresponding author.

Table S2. Bootstrap 95% confidence intervals for network topology metrics

Bootstrap 95% CIs obtained by resampling patients with replacement (200 iterations); in each iteration, the entire network was reconstructed.

Age stratum	Edges	Density	Avg. degree	Clustering coeff.	Modularity
L1 (0–17)	48 [44, 56]	0.034 [0.031, 0.040]	1.78 [1.63, 2.07]	0.202 [0.136, 0.273]	0.73 [0.68, 0.78]
L2 (18–29)	86 [80, 96]	0.041 [0.038, 0.046]	2.65 [2.46, 2.95]	0.284 [0.237, 0.329]	0.68 [0.63, 0.73]
L3 (30–44)	83 [77, 93]	0.033 [0.031, 0.037]	2.34 [2.17, 2.62]	0.192 [0.149, 0.237]	0.57 [0.52, 0.62]
L4 (45–59)	64 [58, 73]	0.030 [0.027, 0.034]	1.94 [1.76, 2.21]	0.259 [0.193, 0.322]	0.61 [0.55, 0.67]
L5 (60+)	60 [54, 70]	0.031 [0.028, 0.036]	1.90 [1.71, 2.22]	0.191 [0.132, 0.261]	0.71 [0.64, 0.78]
All ages	167 [163, 183]	0.060 [0.059, 0.066]	4.45 [4.35, 4.88]	0.414 [0.356, 0.449]	0.53 [0.49, 0.57]

Table S3. Inter-layer network similarity metrics

Layer pair	DeltaCon similarity	Ipsen–Mikhailov distance	JS divergence
L1–L2	0.187	0.351	0.093
L2–L3	0.234	0.283	0.054
L3–L4	0.247	0.265	0.048
L4–L5	0.261	0.242	0.041

The L4–L5 transition was the most stable (DeltaCon = 0.261); L1–L2 was the most dramatic (DeltaCon = 0.187).

Table S4. Disease community membership in the all-age network

Community	Members	N	Description
1	K04.0, K04.4, K04.5, K07.3, K08.1, K08.3, …	18	Restorative/defect cluster
2	K00.0, K00.1, K00.2, K02.1, K02.4, K02.8, K02.9, …	11	Developmental/caries cluster

Community	Members	N	Description
3	K09.0, K09.1, K09.2, K10.2, K10.9, K13.7, …	9	Cyst/mass cluster
4	K01.0, K01.1, K05.2, S02.5, S03.2, T81.0, …	8	Extraction/surgical cluster
5	G90.6, K12.0, K13.0, K14.0, K14.6, L43.9, …	8	Oral mucosal immune cluster
6	K05.0, K05.1, K05.3, K05.5, K05.6, K06.1, K06.8	7	Periodontal cluster
7	C02.9, C03.0, K13.2, K14.9, …	6	Oral neoplasm cluster
8	D11.0, D11.7	2	Parotid cluster

Table S5. Top 10 most frequent two-step progression sequences

Rank	Sequence	No. of patients	Support (%)
1	Dental caries → Tooth defect	16,934	4.58
2	Pulpitis → Tooth defect	16,429	4.44
3	Apical periodontitis → Tooth defect	12,708	3.43
4	Dental caries → Pulpitis	11,961	3.23
5	Dental caries → Apical periodontitis	11,472	3.10
6	Periodontitis → Partial edentulism	10,357	2.80
7	Pulpitis → Apical periodontitis	9,841	2.66
8	Dental caries → Partial edentulism	9,521	2.57
9	Tooth defect → Partial edentulism	8,874	2.40
10	Impacted teeth → Dental caries	8,012	2.16

Table S6. Full temporal precedence analysis

Disease pair	n_both	n_forward	n_reverse	TPR	*p*	Median gap (days)	Type
Pulpitis → Tooth defect	35,366	16,429	4878	0.771	<0.001	27	Pathway
Periapical → Tooth defect	29,758	12,708	5983	0.680	<0.001	21	Pathway
Caries → Tooth defect	39,313	16,934	11,260	0.601	<0.001	131	Pathway
Periodontitis → Edentulism	24,784	10,357	5915	0.636	<0.001	98	Pathway
Caries → Pulpitis	36,696	11,961	10,186	0.540	<0.001	54	Pathway
TMJ → Pulpitis	638	189	323	0.369	<0.001	223	Neg. ctrl
Malocclusion → Pulpitis	4685	1538	1811	0.459	<0.001	178	Neg. ctrl

Table S7. Inter-diagnosis gap distribution for key pathway pairs

Disease pair	N patients	Same-day (%)	<30 days (%)	Median gap (days)
Caries–Pulpitis	36,696	1.6	40.2	54
Pulpitis–Tooth defect	35,366	3.3	41.0	39
Periapical–Tooth defect	29,758	6.1	37.9	53
Caries–Tooth defect	39,313	6.8	28.8	131
Periodontitis–Edentulism	24,784	10.9	34.7	98

Table S8. Gap-stratified temporal precedence ratio

Disease pair	Gap ≥30 d	≥180 d	≥365 d	≥730 d
Pulpitis → Tooth defect	0.771 ***	0.635 ***	0.621 ***	0.642 ***
Caries → Tooth defect	0.601 ***	0.584 ***	0.590 ***	0.617 ***

Disease pair	Gap ≥30 d	≥180 d	≥365 d	≥730 d
Periodontitis → Edentulism	0.636 ***	0.644 ***	0.652 ***	0.681 ***
TMJ → Pulpitis (neg. ctrl)	0.369 ***	0.348 ***	0.312 ***	0.230 ***

*** $p < 0.001$.

Table S9. TPR stratified by visit frequency tertiles

Disease pair	T1 (low)	T2 (mid)	T3 (high)	All
Pulpitis → Tooth defect	0.767 ***	0.827 ***	0.761 ***	0.771 ***
Caries → Pulpitis	0.585 ***	0.556 ***	0.532 ***	0.540 ***
Periodontitis → Edentulism	0.659 ***	0.669 ***	0.630 ***	0.636 ***

Table S10. Time-respecting T-DAG edges for all 167 all-age network edges and head-to-head comparison with the PC-derived DAG

Table S10A reports, for all 167 edges of the all-age comorbidity network, the time-respecting causal direction inferred from patient-level first-diagnosis temporal precedence (TPR-based orientation). Table S10B presents the head-to-head comparison between the time-respecting T-DAG and the cross-sectional PC-derived DAG restricted to the 8 overlapping edges where both methods returned an orientation; the PC algorithm disagreed with the time-respecting direction in 5 of 8 cases (62.5%).

Table S10A. Time-respecting T-DAG: per-edge results for all 167 all-age network edges

source	target	n_both	n_forward	n_reverse	n_concurr	TPR	p_value	RR	p_bonferroni	direction
K07.1	K00.6	7351	2630	2911	1810	0.475	1.68e-04	2.279	0.0281	reverse
K07.1	K07.6	3882	1378	644	1860	0.682	4.49e-61	2.649	7.50e-59	forward
K07.1	K00.1	1699	325	609	765	0.348	1.03e-20	2.143	1.73e-18	reverse
K07.1	K01.0	941	336	131	474	0.719	8.01e-22	4.242	1.34e-19	forward
K07.1	K08.8	148	62	32	54	0.660	2.59e-03	1.582	0.4321	undirected
K07.1	K07.2	118	31	62	25	0.333	1.71e-03	2.529	0.2860	undirected
K07.1	K06.1	80	59	15	6	0.797	2.55e-07	1.812	4.26e-05	forward
K08.1	K08.3	14728	2604	7015	5109	0.271	0.00e+00	2.983	0.00e+00	reverse
K08.1	S02.5	5499	1038	2750	1711	0.274	2.55e-176	2.454	4.26e-174	reverse
K08.1	K04.9	1595	524	748	323	0.412	3.65e-10	1.801	6.09e-08	reverse
K08.1	K00.0	615	241	157	217	0.606	2.98e-05	3.855	4.98e-03	forward
K08.1	S03.2	295	39	88	168	0.307	1.63e-05	2.263	2.73e-03	reverse
K02.9	K00.6	11215	5904	3552	1759	0.624	1.78e-130	2.283	2.97e-128	forward
K02.9	K02.1	2155	823	564	768	0.593	3.72e-12	3.790	6.21e-10	forward
K02.9	K02.8	1055	578	216	261	0.728	6.44e-39	3.539	1.07e-36	forward
K07.3	K04.4	29758	5983	12708	11067	0.320	0.00e+00	1.725	0.00e+00	reverse
K07.3	K04.0	35366	4878	16429	14059	0.229	0.00e+00	2.245	0.00e+00	reverse
K07.3	K08.3	12627	3262	4336	5029	0.429	6.23e-35	1.890	1.04e-32	reverse
K07.3	K04.5	8918	2242	3710	2966	0.377	1.96e-81	1.785	3.27e-79	reverse
K07.3	K02.4	1574	409	589	576	0.410	1.34e-08	1.961	2.24e-06	reverse
K07.3	K03.8	1222	381	436	405	0.466	0.0588	1.626	1.0000	undirected
K07.3	K02.8	650	314	188	148	0.625	2.05e-08	2.240	3.43e-06	forward
K07.3	K03.0	422	223	100	99	0.690	6.35e-12	2.219	1.06e-09	forward

K05.6	K05.3	18787	4799	5698	8290	0.457	1.78e-18	4.853	2.98e-16	reverse
K05.6	K05.1	9049	2261	2397	4391	0.485	0.0479	1.640	1.0000	undirected
K05.6	K03.1	5343	2631	1046	1666	0.716	2.23e-155	1.990	3.73e-153	forward
K05.6	K04.9	3499	1445	535	1519	0.730	3.06e-96	6.325	5.11e-94	forward
K05.6	K06.8	444	156	74	214	0.678	6.78e-08	2.883	1.13e-05	forward
K05.6	K06.1	93	35	23	35	0.603	0.1480	1.943	1.0000	undirected
K01.1	K05.2	6572	1305	1766	3501	0.425	9.08e-17	4.172	1.52e-14	reverse
K01.1	K10.3	490	99	25	366	0.798	1.30e-11	9.424	2.17e-09	forward
K01.1	T81.0	483	104	19	360	0.846	2.22e-15	5.930	3.71e-13	forward
K04.4	K04.0	25138	5354	7132	12652	0.429	3.94e-57	1.828	6.57e-55	reverse
K04.4	K08.3	9303	3133	2649	3521	0.542	2.08e-10	1.651	3.47e-08	forward
K04.4	K04.5	11072	3244	2260	5568	0.589	2.81e-40	3.350	4.70e-38	forward
K04.4	K04.8	1307	316	242	749	0.566	1.97e-03	2.903	0.3294	undirected
K04.4	K04.7	1197	243	283	671	0.462	0.0889	5.058	1.0000	undirected
K04.4	K00.0	330	194	78	58	0.713	1.42e-12	1.621	2.38e-10	forward
K04.4	K04.6	42	18	5	19	0.783	0.0106	3.576	1.0000	undirected
K04.0	K04.5	6611	2403	1398	2810	0.632	2.59e-60	1.585	4.32e-58	forward
K04.0	K02.4	1069	339	195	535	0.635	4.81e-10	1.534	8.03e-08	forward
K04.0	K04.7	671	222	189	260	0.540	0.1144	1.979	1.0000	undirected
K08.3	K04.5	2689	876	898	915	0.494	0.6181	1.524	1.0000	undirected
K08.3	K04.7	292	93	105	94	0.470	0.4344	1.978	1.0000	undirected
K08.3	K00.0	242	143	55	44	0.722	3.23e-10	3.072	5.40e-08	forward
K08.3	T81.0	104	18	4	82	0.818	4.34e-03	2.103	0.7254	undirected
K04.5	K04.8	491	102	138	251	0.425	0.0237	3.131	1.0000	undirected
K04.5	K04.7	307	66	99	142	0.400	0.0125	2.906	1.0000	undirected
K04.5	K00.0	129	70	37	22	0.654	1.84e-03	2.066	0.3073	undirected
K04.5	K02.8	128	81	32	15	0.717	4.55e-06	1.562	7.59e-04	forward
K05.3	K05.1	3801	1449	1039	1313	0.582	2.07e-16	2.135	3.45e-14	forward
K05.3	K03.1	1753	1149	273	331	0.808	6.70e-128	1.881	1.12e-125	forward
K05.3	K04.9	948	515	128	305	0.801	6.81e-56	3.089	1.14e-53	forward
K05.3	K06.8	111	45	28	38	0.616	0.0604	1.804	1.0000	undirected
K05.1	K05.2	1141	510	453	178	0.530	0.0711	1.675	1.0000	undirected
K05.1	K02.1	291	87	130	74	0.401	4.25e-03	1.574	0.7096	undirected
K05.1	K13.0	133	64	45	24	0.587	0.0842	1.669	1.0000	undirected
K05.1	K00.0	136	48	57	31	0.457	0.4351	1.908	1.0000	undirected
K05.1	K02.8	173	82	56	35	0.594	0.0329	1.892	1.0000	undirected
K05.1	K06.8	115	29	28	58	0.509	1.0000	2.061	1.0000	undirected
K05.1	K00.3	78	40	14	24	0.741	5.35e-04	4.019	0.0894	undirected
K05.1	K12.0	77	28	27	22	0.509	1.0000	3.991	1.0000	undirected
K05.1	K06.1	58	26	15	17	0.634	0.1173	4.309	1.0000	undirected
K00.6	K00.1	618	183	333	102	0.355	3.97e-11	3.126	6.63e-09	reverse
K00.6	K02.1	311	121	149	41	0.448	0.1002	2.408	1.0000	undirected
K00.6	K01.0	122	48	47	27	0.505	1.0000	1.599	1.0000	undirected
K00.6	K00.0	96	52	34	10	0.605	0.0662	1.856	1.0000	undirected

K00.6	K07.2	42	10	27	5	0.270	7.63e-03	3.460	1.0000	undirected
K00.6	K00.5	24	17	6	1	0.739	0.0347	3.437	1.0000	undirected
S02.5	K02.4	437	82	200	155	0.291	1.53e-12	3.668	2.55e-10	reverse
S02.5	K03.8	389	96	155	138	0.382	2.37e-04	3.707	0.0395	reverse
S02.5	K00.0	96	59	25	12	0.702	2.66e-04	2.655	0.0445	forward
S02.5	S03.2	127	26	47	54	0.356	0.0186	5.700	1.0000	undirected
S02.5	K00.2	64	6	7	51	0.462	1.0000	5.872	1.0000	undirected
K03.1	K04.9	284	113	125	46	0.475	0.4759	1.779	1.0000	undirected
K03.1	K02.4	227	84	96	47	0.467	0.4124	1.776	1.0000	undirected
K03.1	K03.8	225	87	83	55	0.512	0.8181	2.009	1.0000	undirected
K03.1	K02.8	106	42	45	19	0.483	0.8304	2.214	1.0000	undirected
K03.1	K03.0	92	42	31	19	0.575	0.2416	3.006	1.0000	undirected
K03.1	K05.5	34	13	10	11	0.565	0.6776	3.377	1.0000	undirected
K07.6	K03.0	47	23	5	19	0.821	9.12e-04	2.248	0.1523	undirected
K05.2	K12.1	491	222	166	103	0.572	5.17e-03	2.385	0.8632	undirected
K05.2	K04.9	218	88	67	63	0.568	0.1079	1.727	1.0000	undirected
K05.2	K10.3	39	17	5	17	0.773	0.0169	2.603	1.0000	undirected
K05.2	K12.2	31	6	2	23	0.750	0.2891	3.580	1.0000	undirected
K05.2	T81.0	47	18	13	16	0.581	0.4731	2.710	1.0000	undirected
K05.2	K12.0	54	24	20	10	0.545	0.6516	6.673	1.0000	undirected
K12.1	L43.9	237	77	82	78	0.484	0.7512	3.952	1.0000	undirected
K12.1	K13.7	170	52	36	82	0.591	0.1093	3.963	1.0000	undirected
K12.1	K13.0	127	62	37	28	0.626	0.0154	5.497	1.0000	undirected
K12.1	K13.2	79	30	14	35	0.682	0.0226	6.641	1.0000	undirected
K12.1	K03.0	46	20	17	9	0.541	0.7428	2.504	1.0000	undirected
K12.1	G90.6	135	73	30	32	0.709	2.72e-05	12.114	4.54e-03	forward
K12.1	K10.3	52	2	9	41	0.182	0.0654	4.810	1.0000	undirected
K12.1	K09.8	34	14	14	6	0.500	1.0000	3.502	1.0000	undirected
K12.1	K12.2	24	4	4	16	0.500	1.0000	3.698	1.0000	undirected
K12.1	K14.0	115	37	39	39	0.487	0.9088	12.982	1.0000	undirected
K12.1	K12.0	93	30	26	37	0.536	0.6889	17.530	1.0000	undirected
K12.1	K14.9	21	7	3	11	0.700	0.3438	6.696	1.0000	undirected
K12.1	K14.6	45	11	22	12	0.333	0.0801	10.027	1.0000	undirected
K12.1	C02.9	16	7	2	7	0.778	0.1797	10.899	1.0000	undirected
K12.1	K11.7	23	9	7	7	0.562	0.8036	7.106	1.0000	undirected
K12.1	B37.0	52	13	19	20	0.406	0.3771	31.485	1.0000	undirected
K12.1	D10.3	11	4	1	6	0.800	0.3750	5.994	1.0000	undirected
K12.1	K13.6	10	3	2	5	0.600	1.0000	6.180	1.0000	undirected
K00.1	K01.0	262	64	24	174	0.727	2.37e-05	15.869	3.96e-03	forward
L43.9	K13.0	26	7	11	8	0.389	0.4807	2.647	1.0000	undirected
L43.9	K13.2	111	34	22	55	0.607	0.1409	24.657	1.0000	undirected
L43.9	K14.0	25	14	8	3	0.636	0.2863	6.090	1.0000	undirected
L43.9	K14.6	18	8	7	3	0.533	1.0000	9.199	1.0000	undirected
L43.9	K11.7	15	8	4	3	0.667	0.3877	11.233	1.0000	undirected

L43.9	B37.0	11	4	4	3	0.500	1.0000	11.886	1.0000	undirected
K09.2	K04.8	488	89	119	280	0.428	0.0441	28.526	1.0000	undirected
K09.2	K13.7	63	14	13	36	0.519	1.0000	4.289	1.0000	undirected
K09.2	K04.7	36	6	8	22	0.429	0.7905	2.769	1.0000	undirected
K09.2	K09.0	206	37	48	121	0.435	0.2780	59.147	1.0000	undirected
K09.2	K10.2	11	6	2	3	0.750	0.2891	5.278	1.0000	undirected
K09.2	K10.9	138	16	31	91	0.340	0.0400	44.141	1.0000	undirected
K09.2	K09.1	29	9	5	15	0.643	0.4240	47.209	1.0000	undirected
K09.2	D10.3	17	4	5	8	0.444	1.0000	29.803	1.0000	undirected
K09.2	D11.7	18	7	5	6	0.583	0.7744	60.774	1.0000	undirected
K09.2	K09.9	13	6	3	4	0.667	0.5078	32.030	1.0000	undirected
K04.8	K04.7	132	26	18	88	0.591	0.2912	9.332	1.0000	undirected
K04.8	K09.0	42	8	6	28	0.571	0.7905	8.451	1.0000	undirected
K04.8	K10.9	31	1	2	28	0.333	1.0000	7.362	1.0000	undirected
K02.4	K03.8	662	158	146	358	0.520	0.5282	24.152	1.0000	undirected
K02.4	K05.5	13	9	4	0	0.692	0.2668	4.413	1.0000	undirected
K03.8	K03.0	24	12	7	5	0.632	0.3593	3.031	1.0000	undirected
K03.8	K05.5	15	5	3	7	0.625	0.7266	5.812	1.0000	undirected
K13.7	K13.0	33	2	2	29	0.500	1.0000	4.728	1.0000	undirected
K13.7	K09.0	18	4	6	8	0.400	0.7539	4.634	1.0000	undirected
K13.7	K13.2	64	8	18	38	0.308	0.0755	18.627	1.0000	undirected
K13.7	K06.8	53	6	1	46	0.857	0.1250	10.924	1.0000	undirected
K13.7	K10.9	33	6	7	20	0.462	1.0000	10.359	1.0000	undirected
K13.7	K11.6	66	6	5	55	0.545	1.0000	22.198	1.0000	undirected
K13.7	K09.8	64	5	7	52	0.417	0.7744	24.566	1.0000	undirected
K13.7	K14.0	22	5	5	12	0.500	1.0000	7.470	1.0000	undirected
K13.7	K14.9	35	3	3	29	0.500	1.0000	42.621	1.0000	undirected
K13.7	C02.9	31	12	2	17	0.857	0.0129	89.979	1.0000	undirected
K13.7	D10.3	18	3	4	11	0.429	1.0000	37.029	1.0000	undirected
K13.7	K13.6	17	1	4	12	0.200	0.3750	40.023	1.0000	undirected
K13.7	C03.0	10	3	0	7	1.000	0.2500	51.679	1.0000	undirected
K13.7	K09.9	12	3	1	8	0.750	0.6250	33.902	1.0000	undirected
K02.1	K02.8	34	20	8	6	0.714	0.0357	3.280	1.0000	undirected
K04.7	K12.2	11	1	0	10	1.000	1.0000	6.609	1.0000	undirected
K04.7	K04.6	11	2	1	8	0.667	1.0000	29.195	1.0000	undirected
K01.0	K09.0	18	6	2	10	0.750	0.2891	6.356	1.0000	undirected
K13.0	K11.6	19	0	3	16	0.000	0.2500	10.803	1.0000	undirected
K13.0	K09.8	28	4	5	19	0.444	1.0000	18.330	1.0000	undirected
K13.0	K12.0	15	7	4	4	0.636	0.5488	14.581	1.0000	undirected
K09.0	K10.9	20	3	6	11	0.333	0.5078	20.141	1.0000	undirected
K13.2	K06.8	14	2	1	11	0.667	1.0000	9.654	1.0000	undirected
K13.2	K14.0	15	6	7	2	0.462	1.0000	17.491	1.0000	undirected
K13.2	C02.9	21	8	2	11	0.800	0.1094	186.157	1.0000	undirected
K06.8	K06.1	40	4	8	28	0.333	0.3877	85.864	1.0000	undirected

K11.6	K09.8	57	7	7	43	0.500	1.0000	85.387	1.0000	undirected
G90.6	K14.0	39	4	25	10	0.138	1.04e-04	46.625	0.0173	reverse
G90.6	K14.6	41	8	27	6	0.229	1.88e-03	108.984	0.3137	undirected
G90.6	B37.0	10	2	6	2	0.250	0.2891	51.466	1.0000	undirected
K09.8	K14.0	10	3	6	1	0.333	0.5078	14.879	1.0000	undirected
K14.0	K14.6	45	22	13	10	0.629	0.1755	150.939	1.0000	undirected
K14.0	K11.7	17	8	3	6	0.727	0.2266	76.694	1.0000	undirected
K14.0	B37.0	12	1	5	6	0.167	0.2188	77.895	1.0000	undirected
K14.0	K14.1	10	6	2	2	0.750	0.2891	72.182	1.0000	undirected
K14.9	C02.9	12	4	0	8	1.000	0.1250	364.037	1.0000	undirected
K14.6	K11.7	21	8	6	7	0.571	0.7905	199.000	1.0000	undirected
K11.9	D11.0	14	4	2	8	0.667	0.6875	1060.662	1.0000	undirected

Table S10B. Head-to-head comparison between time-respecting T-DAG and PC-derived DAG (8 overlapping edges)

pair_a	pair_b	time_direction	pc_direction	agree	TPR	n_both
K08.1	K08.3	K08.3 → K08.1	K08.3 → K08.1	True	0.271	14728
K07.3	K04.4	K04.4 → K07.3	K07.3 → K04.4	False	0.320	29758
K07.3	K04.0	K04.0 → K07.3	K07.3 → K04.0	False	0.229	35366
K07.3	K08.3	K08.3 → K07.3	K08.3 → K07.3	True	0.429	12627
K05.6	K05.3	K05.3 → K05.6	K05.3 → K05.6	True	0.457	18787
K04.4	K04.0	K04.0 → K04.4	K04.4 → K04.0	False	0.429	25138
K04.4	K08.3	K04.4 → K08.3	K08.3 → K04.4	False	0.542	9303
K04.4	K04.5	K04.4 → K04.5	K04.5 → K04.4	False	0.589	11072

"agree" indicates whether the PC-derived edge direction matches the T-DAG temporal-precedence direction. For non-matching edges, the PC orientation reverses the clinically expected progression direction supported by the temporal precedence analysis. TPR = temporal precedence ratio.

Supplementary Figures

Figure S1. Topology bar charts across age strata

Bar-chart comparisons of topological metrics (edges, density, avg. degree, clustering coefficient, modularity) across five age strata.

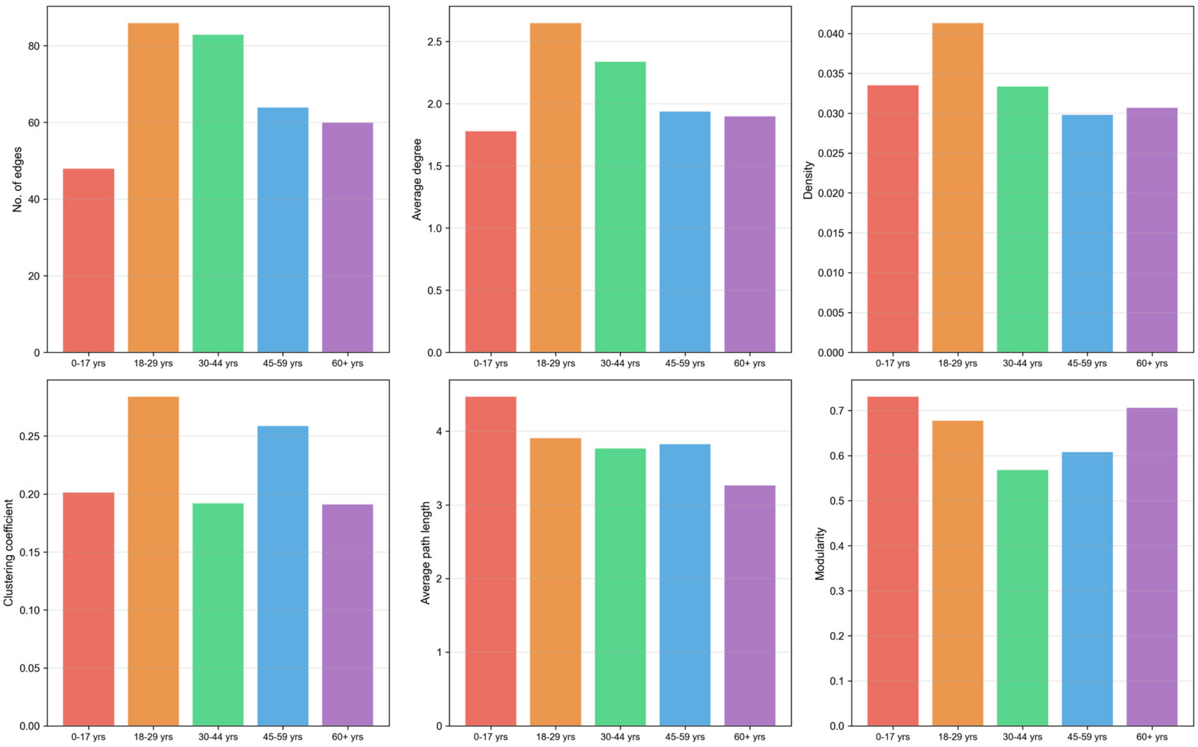

Figure S1

Figure S2. Community structure visualization

Louvain community structure in the all-age comorbidity network. Node color indicates community assignment.

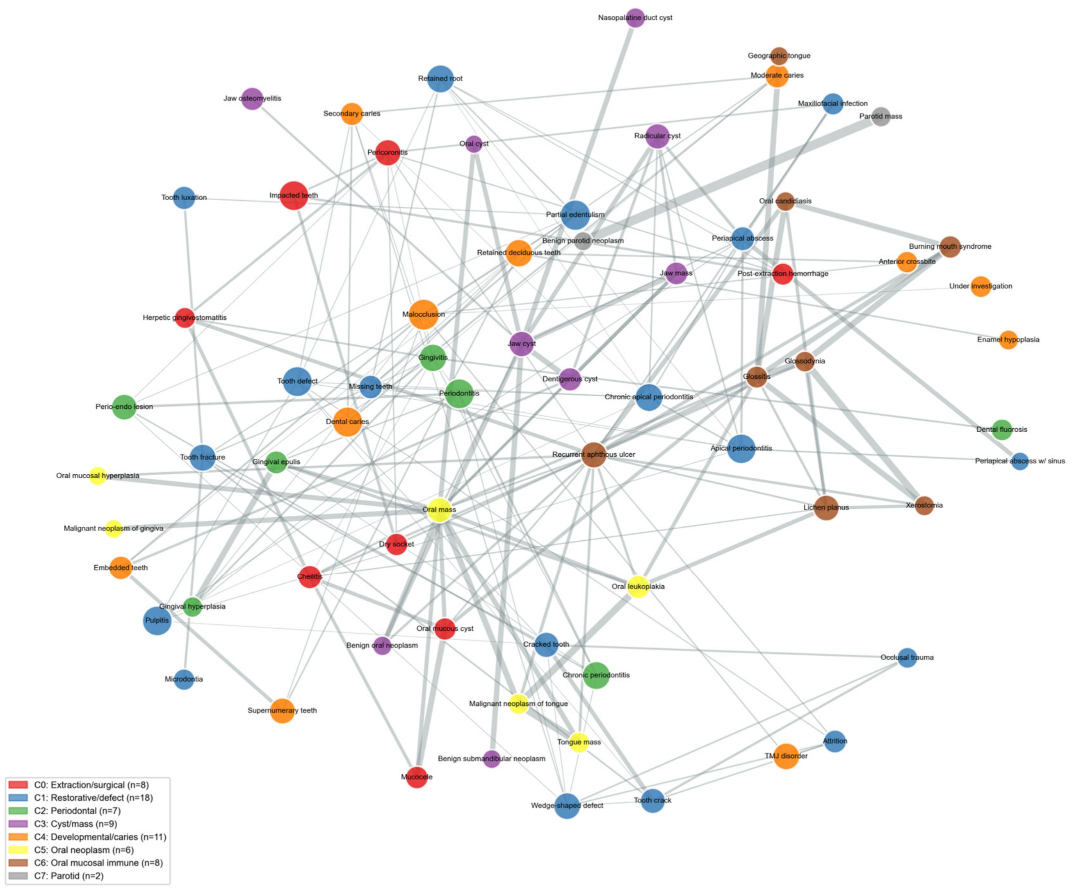

Figure S2

Figure S3. Community stability heatmap

Heatmap of community assignment stability across age strata for each disease node.

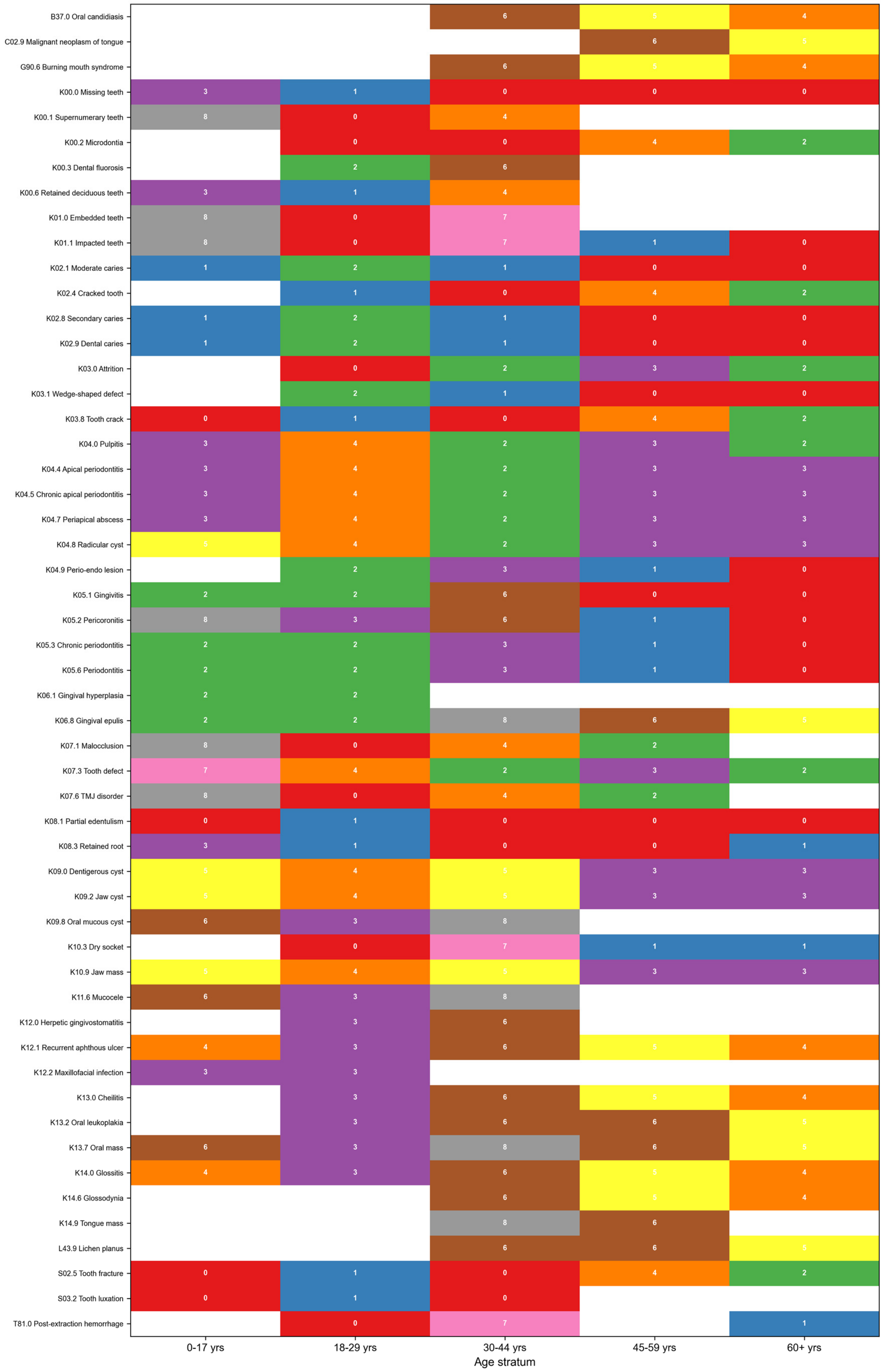

Figure S3

Figure S4. Bifurcation path analysis for hub diseases

Bifurcation paths from five hub diseases showing downstream disease branching probabilities.

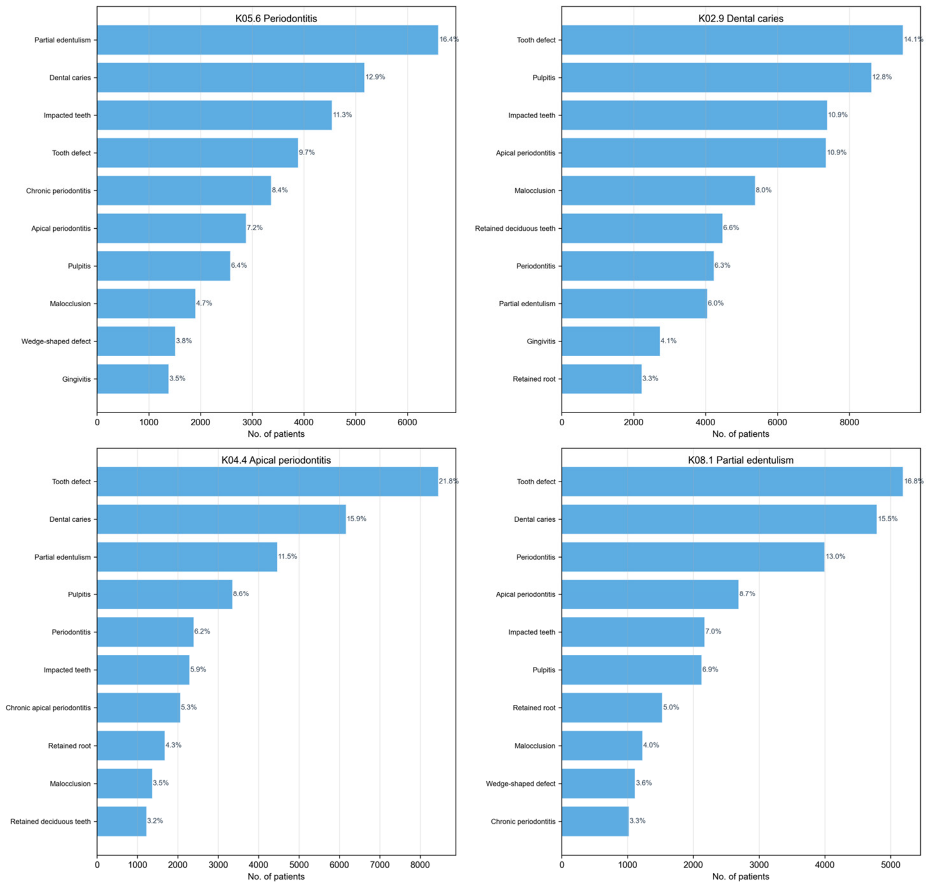

Figure S4

Figure S5. Sensitivity analysis panels

Eight-dimensional sensitivity analysis panels (S1–S8).

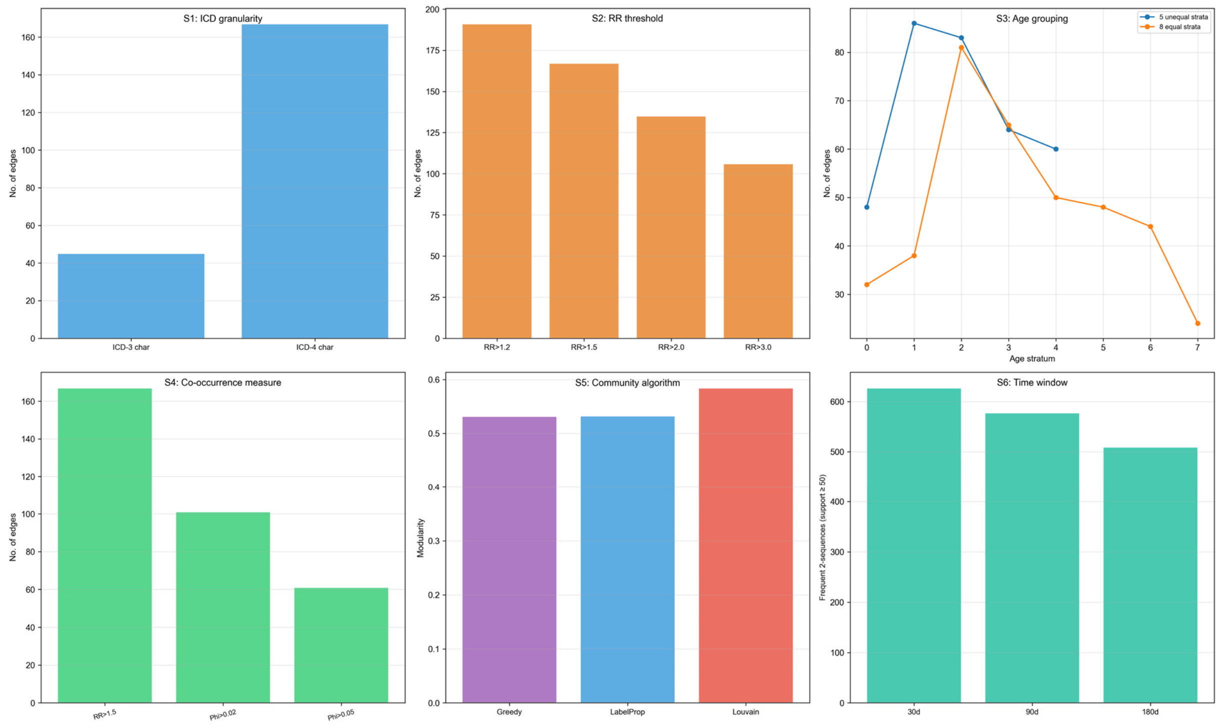

Figure S5

Figure S6. Inter-layer similarity analysis

(a) DeltaCon similarity heatmap; (b) Ipsen–Mikhailov spectral distance; (c) JS divergence; (d) normalized distance curves.

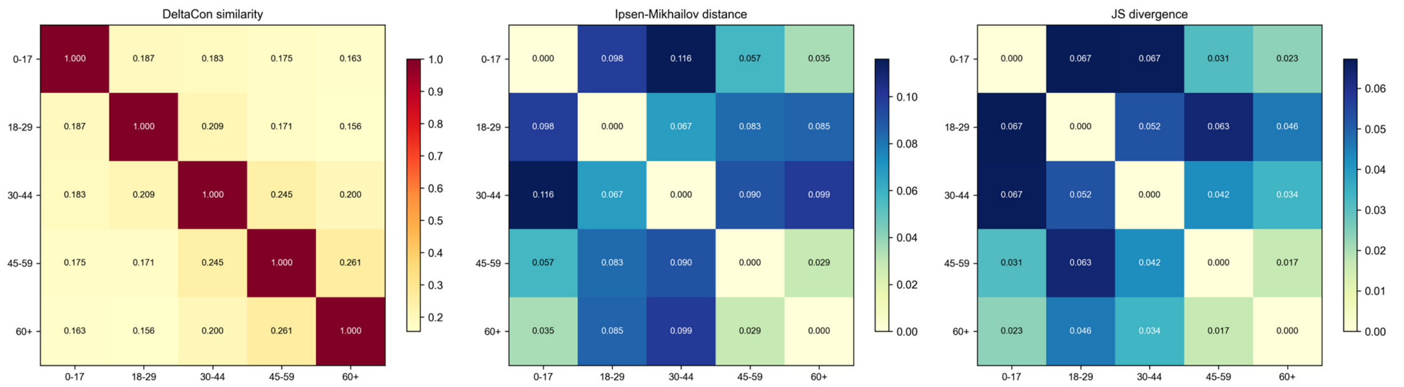

Figure S6

Figure S7. Sex-stratified network comparison

Male and female comorbidity network comparison: topology metrics, centrality rankings, and sex-specific edges.

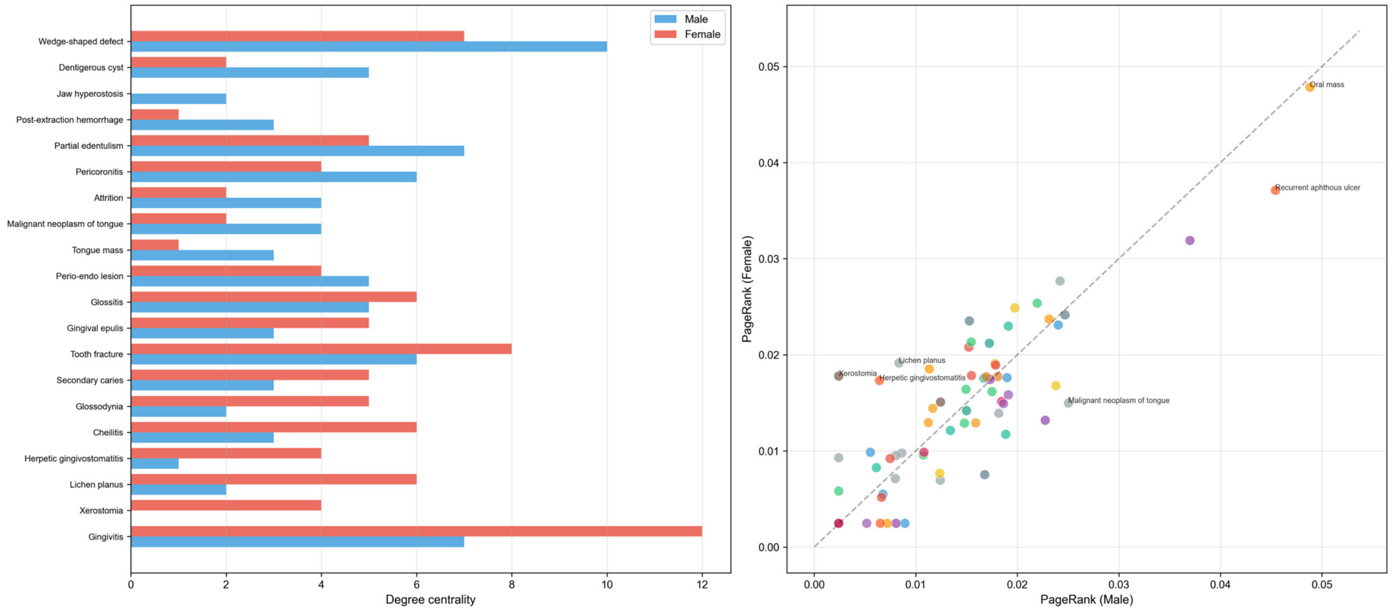

Figure S7

Supplementary Results

S9. Quasi-causal sensitivity analyses

S9.1 Inter-diagnosis gap stratification

The proportion of same-day co-diagnoses was low for pathway pairs (caries–pulpitis: 1.6%; pulpitis–tooth defect: 3.3%), and the majority of co-occurring patients had gaps exceeding 30 days (**Table S7**). TPR for pathway pairs remained robust at all gap thresholds (**Table S8**), while negative controls showed progressively reversed directionality, confirming that the observed temporal patterns are not artifacts of the single-code-per-visit constraint.

S9.2 Visit frequency stratification

TPR patterns were consistent across visit frequency tertiles (**Table S9**), ruling out Berkson's bias.

S9.3 Constraint-based causal discovery

The PC algorithm (10,000-patient subsample, $\alpha = 0.001$) yielded 35 edges. The FCI algorithm identified only 4 confidently directed edges. IDA-based causal effect estimates were consistent with Cox regression (e.g., pulpitis → tooth defect: adjusted OR = 2.00). However, the PC-derived DAG reversed the direction of caries and pulpitis, placing them as terminal sinks — demonstrating that constraint-based DAG learning on cross-sectional binary data identifies conditioning structure, not temporal causation. *[Figure originally numbered S8 was moved to the main text as Figure 3.]*

S9.4 Department transfer network

A patient longitudinal transfer network among 17 clinical departments showed that the Department of Operative Dentistry and Endodontics was the largest net outflow (source), while the Department of Oral and Maxillofacial Surgery was the largest net inflow (sink). This pattern corroborates the disease-level progression chain: pulp/periapical lesions → tooth defect → surgical management.
